# Photocatalytic CO_2_ Reduction Using Various Heteroleptic Diimine-Diphosphine Cu(I) Complexes as Photosensitizers

**DOI:** 10.3389/fchem.2019.00288

**Published:** 2019-04-30

**Authors:** Yasuomi Yamazaki, Takayuki Onoda, Jun Ishikawa, Shota Furukawa, Chinatsu Tanaka, Tomoya Utsugi, Taro Tsubomura

**Affiliations:** Department of Materials and Life Science, Seikei University, Musashino-shi, Japan

**Keywords:** CO_2_ reduction, photocatalytic reaction, Cu(I) complexes, redox-photosensitizer, diphosphine ligands

## Abstract

The development of efficient redox-photosensitizers based on the earth-abundant metal ions as an alternative toward noble- and/or rare-metal based photosensitizers is very desirable. In recent years, heteroleptic diimine-diphosphine Cu(I) complexes have been well investigated as one of the most remarkable candidates because of their great potentials as efficient photosensitizers. Here, we investigated the effects of the structure of the diphosphine ligands on the photosensitizing abilities using a series of Cu(I) complexes bearing 2,9-dimethyl-4,7-diphenyl-1,10-phenanthroline (dmpp) and various diphosphine ligands in order to explore the suitable structure for the photosensitizing reactions. The number of methylene chains between the two phosphorous atoms in the diphosphine ligands was systematically changed from two to four, and the relationship between the length of the carbon chains and the photosensitizing abilities were investigated by conducting photocatalytic CO_2_ reduction with the Cu(I) complexes as photosensitizers. Turnover frequencies of the CO_2_ reduction drastically increased with increasing the length of the carbon chains. The systematic study herein reported suggests that the large P-Cu-P angles should be one of the most important factors for enhancing the photosensitizing abilities.

## Introduction

In the last few decades, photocatalytic CO_2_ reduction has been widely investigated due to emerging concerns about the serious environmental problems, e.g., global warming and depletion of carbon and energy resources. Metal complexes have played an important role both as photosensitizers and as CO_2_ reduction catalysts in this research area, owing to their suitable photophysical and photochemical properties, high reaction selectivity, and flexibility in the molecular design (Morris et al., 2009; Windle and Perutz, 2012; Sahara and Ishitani, 2015; Yamazaki et al., 2015; Kuramochi et al., 2018). Noble- and/or rare-metal complexes in particular have shown quite high efficiency and durability. For instance, the photocatalytic systems constructed with a Re(I) tricarbonyl complex as a CO_2_-reduction catalyst and a ring-shaped Re(I) multinuclear complex as a photosensitizer, can trigger CO_2_ reduction with tremendously high quantum yield (Φ_CO_ = 82%) (Morimoto et al., 2013; Rohacova and Ishitani, 2016, 2017). Another example is a system using a Ru(II)-Re(I) multinuclear complexes, (Gholamkhass et al., 2005; Sato et al., 2007; Koike et al., 2009; Tamaki et al., 2012, 2013a; Kato et al., 2015; Ohkubo et al., 2016; Tamaki and Ishitani, 2017; Yamazaki and Ishitani, 2018) which shows both high efficiency and durability, i.e., up to 50% of Φ_CO_ and more than 3,000 of the turnover number (TON_CO_) (Tamaki et al., 2013a; Tamaki and Ishitani, 2017; Yamazaki and Ishitani, 2018). Though the fundamental researches using noble- and/or rare-metals must be quite important in order to fully understand the reaction mechanism of the CO_2_ reduction and to explore strategies of molecular design for efficient components in photocatalytic systems, in the future, such metals which lie under the ground in small amounts should be replaced by earth-abundant elements because the amount of emission of CO_2_ relating to consumption of fossil fuels is quite large and is increasing year by year (Takeda et al., 2016a).

In recent years, heteroleptic diimine-diphosphine Cu(I) complexes have been reported as an alternative toward noble- and/or rare-metal based photosensitizers (Takeda et al., 2016b, 2018; Heberle et al., 2017; Rosas-Hernández et al., 2017; McCullough et al., 2018; Zhang et al., 2018). Some of them show not only high efficiency but also high durability equal to or higher than those of noble- and/or rare-metal complexes, and the highest Φ_CO_ and TON_CO_ in the photocatalytic systems for CO_2_ reduction using Cu(I) photosensitizers were 57% and >1,300, respectively (Takeda et al., 2018). These reports clearly indicate that Cu(I) complexes should be powerful candidates not just as an alternative toward photosensitizers based on noble- and/or rare-metal ions, but as one of the most efficient photosensitizers. Therefore, further investigation using series of Cu(I) complexes to clarify the relationship between the molecular structure and photosensitizing abilities should be useful to explore the suitable molecular design for the efficient Cu(I)-complex photosensitizers.

**Chart 1:**
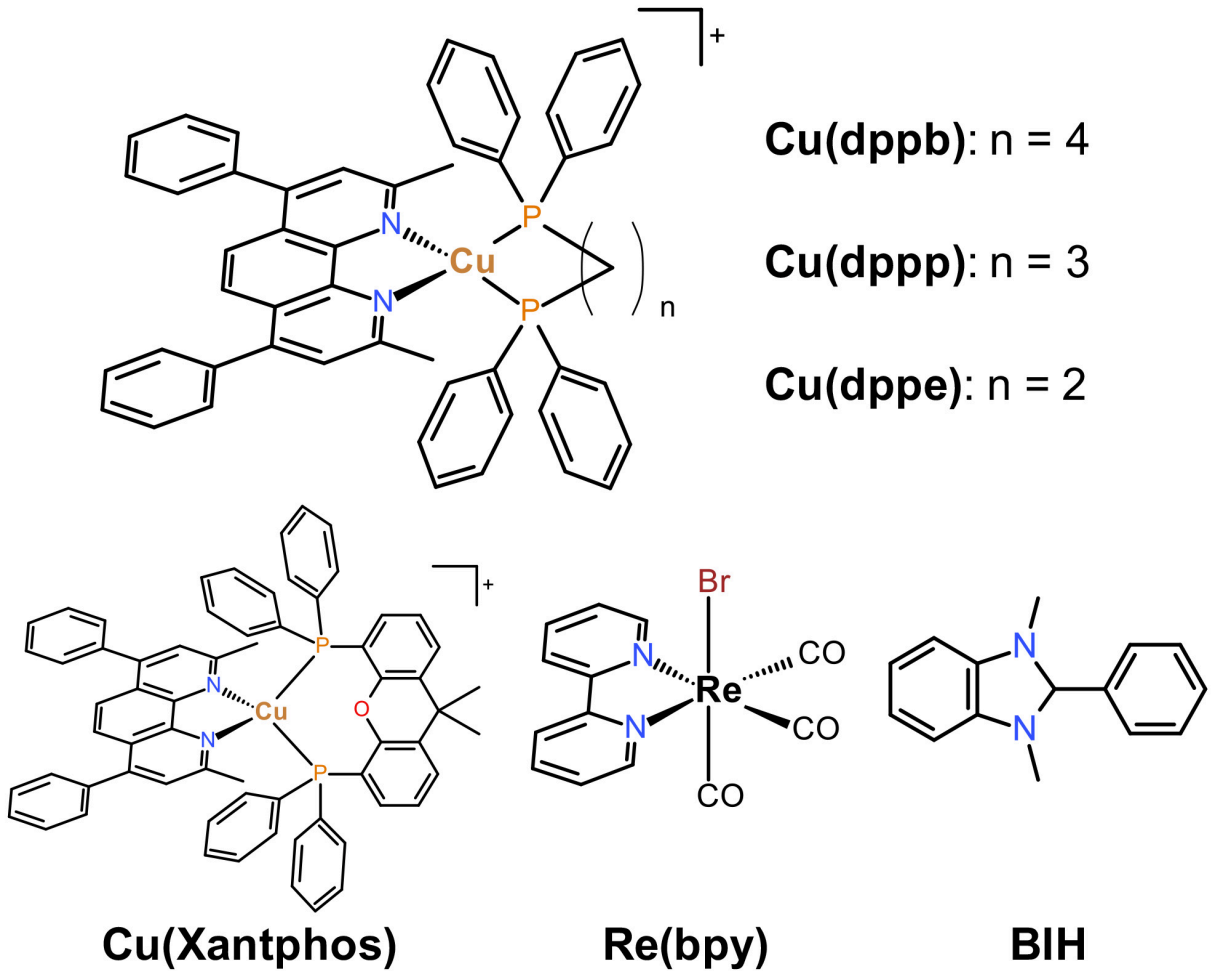
Structures and abbreviations of the Cu(I) complexes, the Re(I) complex and the electron donor used in this study.

We previously reported photophysical properties of series of diimine-diphosphine Cu(I) complexes bearing 1,10-phenanthroline derivatives and various bidentate phosphine ligands (Saito et al., 2006; Tsubomura et al., 2015; Nishikawa et al., 2017). In particular, a series of the Cu(I) complexes having 2,9-dimethyl-4,7-diphenyl-1,10-phenanthroline (dmpp) showed high molar extinction coefficients and emission quantum yields compared with those without phenyl groups at 4,7 positions (Tsubomura et al., 2015). The photophysical properties of the dmpp complexes were strongly affected by the structure of the bidentate phosphine ligands; wavelength of both absorption bands and emission maxima were blue-shifted and emission lifetimes became longer with increasing the length of the methylene chains between the two phosphorous atoms, likely due to the difference in the bite angles of the chelate-phosphine ligands (P-Cu-P angles). The strong absorption abilities and long emission lifetimes should be useful not only as photo-luminescent materials but also as photosensitizers for photochemical reactions. Therefore, in this study, we examined the effects of diphosphine ligands in detail on the photosensitizing abilities using a series of Cu(I) complexes bearing dmpp ligands shown in Chart 1, i.e., dmpp complexes with 3 types of diphosphine ligands with different length of carbon chains: dppe (1,2-bis(diphenylphosphino)ethane), dppp (1,3-bis(diphenylphosphino)propane), and dppb (1,4-bis(diphenylphosphino)butane). The dmpp complex bearing Xantphos (4,5-Bis(diphenylphosphino)-9,9-dimethylxanthene), which is often used as a diphosphine ligand of Cu(I) photosensitizers, was also investigated for comparison. The systematic study herein reported suggests that the P-Cu-P angles should be one of the most important factors which determines the photosensitizing abilities.

## Results and Discussion

### Photocatalytic CO_2_ Reduction Using Cu(I) Complexes as Photosensitizers

**Figure 1:**
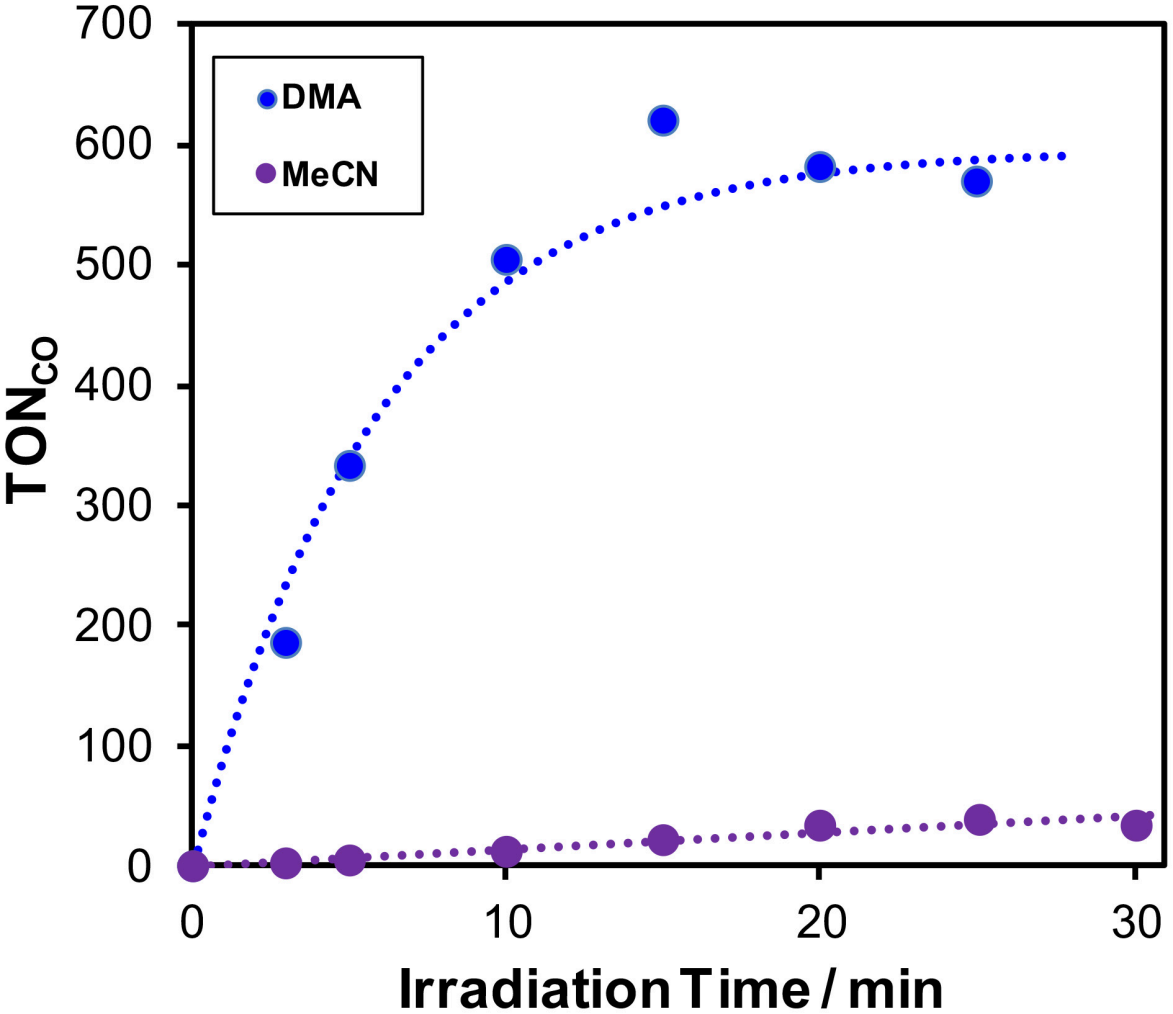
Time courses of the TON of CO formation during photocatalytic reactions (λ_ex_ > 370 nm) using a mixture of MeCN and TEOA (4:1 v/v, purple) or a mixture of DMA and TEOA (4: 1 v/v, blue) containing 0.5 mM **Cu(dppb)**, 0.05 mM **Re(bpy)**, and 0.1 M BIH.

In a typical run of photocatalytic reactions, a mixed solution of MeCN-TEOA (TEOA = triethanolamine, 4:1 v/v) containing **Cu(dppb)** (0.5 mM) as a photosensitizer, Re(2,2'-bipyridine)(CO)_3_Br (**Re(bpy)**, 0.05 mM) as a CO_2_-reduction catalyst and 1,3-dimethyl-2-phenyl-2,3-dihydro-1*H*-benzo[*d*]imidazole (BIH, 0.1 M) as an electron donor was irradiated under a CO_2_ atmosphere using a high-pressure mercury-lamp equipped with a UV-cut filter (>370 nm, Figure 1). **Re(bpy)** is well known not only as a photocatalyst for CO_2_ reduction (Hawecker et al., 1983, 1986; Kutal et al., 1985) but also as a CO_2_-reduction catalyst in photocatalytic systems, which can produce CO with high selectivity; (Gholamkhass et al., 2005; Sato et al., 2007; Kiyosawa et al., 2009; Koike et al., 2009; Morris et al., 2009; Tamaki et al., 2012, 2013a,b; Kou et al., 2014; Kato et al., 2015; Ohkubo et al., 2016). Therefore, we measured the gaseous products of the photocatalysis using gas-chromatography. CO was selectively produced and no hydrogen was detected over 30-min irradiation. TON_CO_ after 30-min irradiation was 40. Though various other CO_2_-reduction catalysts, i.e., Re(4,4'-dimethyl-2,2'-bipyridine)(CO)_3_Br, Re(1,10-phenanthroline)(CO)_3_Br, and Ru(6,6'-dimethyl-2,2'-bipyridine)(CO)_2_Cl_2_, were also used instead of **Re(bpy)**, the system using **Re(bpy)** showed the highest TON_CO_ in this reaction condition (Figure S1).

In the case of using a mixed solvent of DMA-TEOA (DMA = *N*,*N*-dimethylacetamide, 4:1 v/v) instead of that of MeCN-TEOA (4:1 v/v), TON_CO_ increased and reached 580 after 25-min irradiation (Figure 1). The turnover frequency (TOF), which was determined from the slopes of the fitting curves of the time course in the initial stage of the photocatalysis, was relatively high (65 min^−1^, Figure S2). This result suggests that DMA should be more preferable solvent for the efficient photocatalytic reaction using **Cu(dppb)** and **Re(bpy)**. In the absence of **Cu(dppb)**, TON_CO_ after 1-h irradiation were 20; thus, the photocatalysis by only **Re(bpy)** should be not sufficient under the condition. In addition, in the absence of **Re(bpy)** or BIH, TON_CO_ were 0 or 7, respectively. These results clearly indicate that **Cu(dppb)** has relatively high photosensitizing ability and promoted CO_2_ reduction on **Re(bpy)** using BIH as an electron donor. From these results, we concluded the photocatalyses using **Re(bpy)** as a CO_2_-reduction catalyst and a mixed solvent of DMA-TEOA as a reaction solvent are suitable for the investigation to clarify the photosensitizing abilities of the series of the Cu(I) complexes shown in Chart 1.

**Figure 2:**
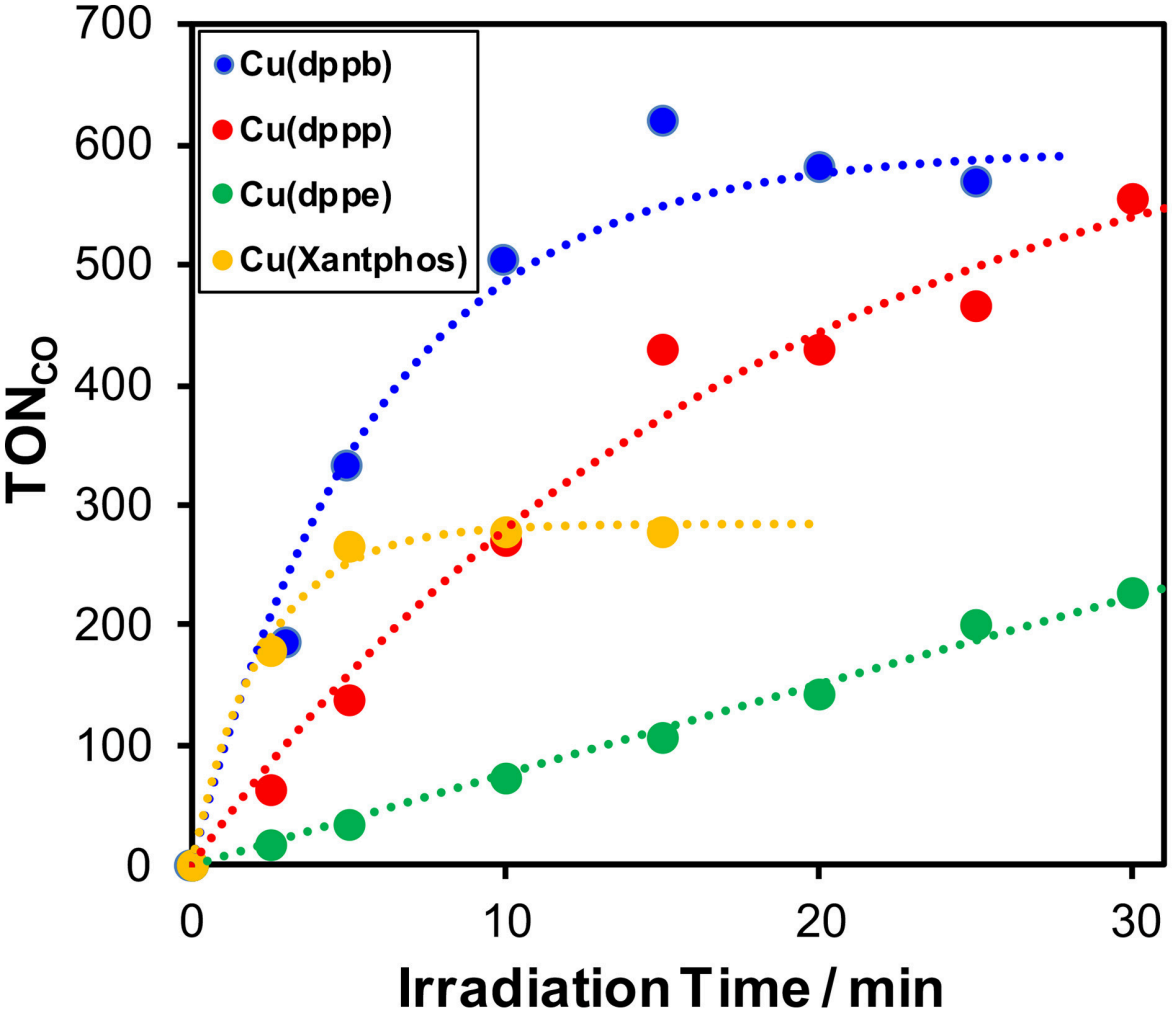
Time courses of the TON of CO formation during photocatalytic reactions (λ_ex_ > 370 nm) using a mixture of DMA and TEOA (4:1 v/v) containing 0.05 mM **Re(bpy)**, 0.1 M BIH, and various Cu(I) complexes [blue: **Cu(dppb)**, red: **Cu(dppp)**, green: **Cu(dppe)**, orange: **Cu(Xantphos)**]. The concentration of each Cu(I) complex is 0.5 mM.

Mixed solutions of DMA-TEOA (4:1 v/v) containing **Re(bpy)** (0.05 mM), BIH (0.1 M), and various Cu(I) complexes (0.5 mM) were shined under the same condition described above. Figure 2 shows the time-courses of the CO production and both TON_CO_ and TOF are summarized in Table 1 and Figure S2. Interestingly, both TON_CO_ and TOF strongly depended on the structure of the diphosphine ligands. When comparing **Cu(dppb)**, **Cu(dppp)**, and **Cu(dppe)**, TOF drastically increased with increasing the length of the methylene chains between the two phosphorous atoms in the diphosphine ligands. In the case using **Cu(Xantphos)**, TOF was quite high and almost equal to that of **Cu(dppb)**. In contrast, TON_CO_ of **Cu(Xantphos)** was low, and the time course of CO formation reached plateau within 5-min of irradiation. The absorption spectral changes of the reaction solutions after irradiation are shown in Figure S3. In all cases, the metal-to-ligand charge transfer (MLCT) absorption bands of the Cu(I) complexes decreased gradually; therefore, one of the main reasons for the decreasing of the reaction rate with increasing the irradiation time should be the photo-decomposition of the Cu(I) complexes. Curiously, in the case using **Cu(Xantphos)**, the photocatalytic CO formation stopped within 5 min even though the MLCT absorption band was obviously observed in the absorption spectrum of the reaction solution after 5-min irradiation. Though the reason for the low TON_CO_ when using **Cu(Xantphos)** is not clear at this stage, the degradation of **Re(bpy)** possibly proceeded more rapidly than the other cases. As described later, **Cu(Xantphos)** showed quite small absorption in the longer wavelength region (>430 nm) compared to the other Cu(I) complexes. It has been recently reported that the irradiation with short wavelength light (<450 nm) drastically lowered the photo-stability of Re(I) complexes; (Lang et al., 2019) thus, it is expected that **Re(bpy)** could absorb the irradiation light in the longer wavelength region, e.g., the emission line at 436 nm from the high-pressure mercury lamp, more frequently and photo-decomposition of **Re(bpy)** proceeded rapidly. Since the analysis of the Re(I) complexes, e.g., measurement of FT-IR spectra of the reaction solutions, was difficult because of the low concentration of the Re(I) complexes, further investigation is now undergoing.

**Table 1:** Photocatalytic properties using the mixed system of the Cu(I) photosensitizers and **Re(bpy)**^a^.

**Photosensitizer**	**TON^b^**		**TOF^c^/min^−1^**	**η_**q**_/%**	**$\Phi_{{\text{CO}}^{\text{g}}}$/%**
	**CO**	**H_**2**_**			
**Cu(dppb)**	580	n.d.	65	99^e^	37
	40^d^	n.d.^d^	1.4^d^	99^e^	–
**Cu(dppp)**	560	n.d.	27	99^f^	–
**Cu(dppe)**	230	n.d.	7.3	98^f^	–
**Cu(Xantphos)**	240	n.d.	72	99^f^	–

a *A CO_2_-saturated DMA-TEOA (4:1 v/v) mixed solution containing the Cu(I) photosensitizer (0.5 mM), **Re(bpy)** (0.05 mM), and BIH (0.1 M) was irradiated*.

b *Maximum TON_CO_ within 30-min irradiation calculated as [product (mol)]/[added **Re(bpy)** (mol)]*.

c *TOF was determined by the slopes of the fitting curves over 5 min (Figure S2)*.

d *A mixed solution of MeCN-TEOA (4:1 v/v) was used instead*.

e *Quenching fractions of emission from Cu(I) photosensitizers by BIH determined from Stern-Volmer analyses in a mixture of MeCN-TEOA (4:1 v/v)*.

f *Quenching fractions of emission from Cu(I) photosensitizers by BIH determined from Stern-Volmer analyses in a mixture of DMA-TEOA (4:1 v/v)*.

g *Quantum yield of CO production calculated as [CO (mol)]/[absorbed photon (einstein)] (λ_ex_ = 430 nm, light intensity: 1.0 × 10^−8^ einstein/s)*.

In order to precisely evaluate the efficiency of CO_2_ reduction, the quantum yield for CO formation (Φ_CO_) was determined using the system of **Cu(dppb)** as a typical example. BIH is well known as a two-electron donor, which can induce the second-electron injection even to ground-state of photosensitizers and/or CO_2_-reduction catalysts owing to the quite strong reducing power of the radical species produced after oxidation by the excited state of photosensitizers and subsequent deprotonation by TEOA (Equation 1); (Tamaki et al., 2013a). Thus, the maximum Φ_CO_ of this system should be 100%. A mixture of DMA and TEOA (4:1 v/v) containing 0.5 mM **Cu(dppb)**, 0.05 mM **Re(bpy)**, and 0.1 M BIH was irradiated with 430-nm monochromic light. CO was linearly produced over 1-h irradiation, and Φ_CO_ was determined to be 37% from the slopes of the fitting curves (Figure S4). This value is relatively high among the photocatalytic systems for CO_2_ reduction using Cu(I)-complex photosensitizers.

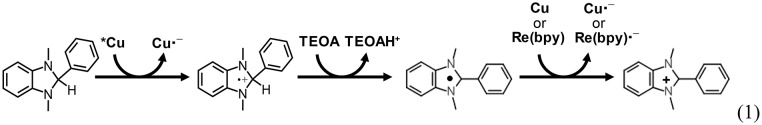



### Properties of Cu(I) Complexes

#### Structure of Cu(I) Complexes in Solutions

**Figure 3:**
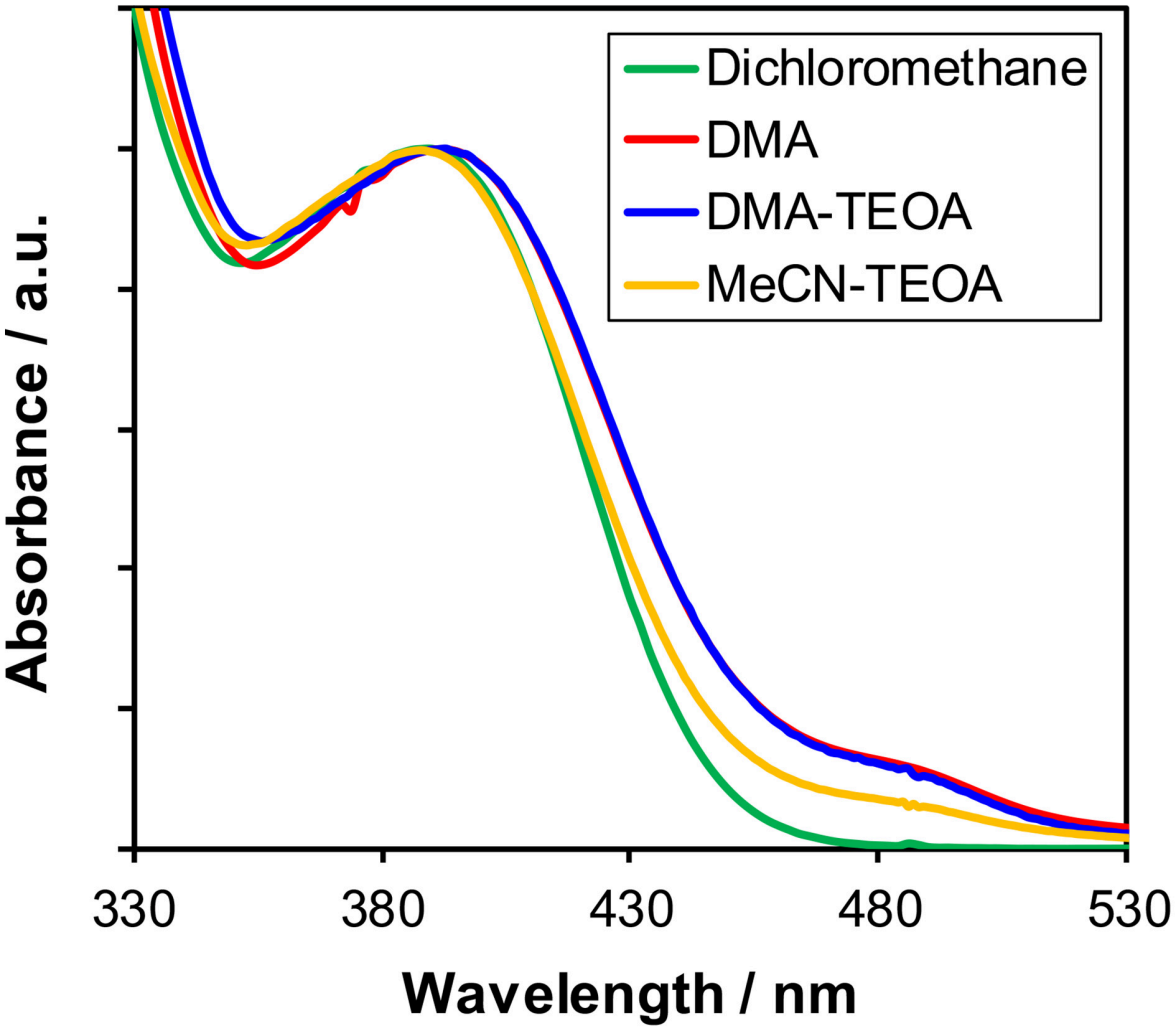
UV-Vis absorption spectra of **Cu(dppb)** in various solutions: dichloromethane (green), DMA (red), a mixture of DMA-TEOA (4:1 v/v, blue), and a mixture of MeCN-TEOA (4:1 v/v, orange).

As described above, the Cu(I) complexes, in particular **Cu(dppb)**, showed relatively high photosensitizing ability. In order to clarify the reason for the difference in the photosensitizing abilities of the Cu(I) complexes, we firstly investigated the molecular structure of the Cu(I) complexes in the reaction solutions in detail because it is known that Cu(I) complexes often cause structural changes in solutions due to the ligand-exchange reactions. Figure 3 illustrates the UV-vis absorption spectra of **Cu(dppb)** in various solutions, i.e., dichloromethane, DMA, a mixture of DMA-TEOA and that of MeCN-TEOA. The shapes of the spectra at around 330–450 nm were similar. Except the spectrum in dichloromethane, small absorption bands were observed at around 450–530 nm. This is probably due to the formation of [Cu(dmpp)_2_]^+^-type complex produced by the disproportionation reaction after dissolving in solvents having coordination ability (Kaeser et al., 2013). Since the molar extinction coefficient of [Cu(dmpp)_2_]^+^ was large (ε_484_ = 10,300 in a DMA-TEOA mixed solution, Figure S5), the amount of the [Cu(dmpp)_2_]^+^ should not be large. ^1^H NMR analyses of **Cu(dppb)** using CD_2_Cl_2_ or CD_3_CN also revealed that the structure in CD_3_CN is almost identical to that in CD_2_Cl_2_, though a few mol-percent of [Cu(dmpp)_2_]^+^ were observed. The other complexes also showed similar spectrum-pattern in both absorption spectra and ^1^H NMR spectra regardless of solvents (Figure S6). Therefore, the main species should be the diimine-diphosphine type complexes even in the reaction solutions for the photocatalytic reactions, and the solvent molecules do not strongly affect the structure and the electronic properties of the ground state of the Cu(I) complexes.

We previously clarified that **Cu(dppb)** is dimerized by bridging with two bidentate phosphine ligands in the solid state using single crystal X-ray structure analyses. The dimerization might be a reason for the higher photosensitizing ability of **Cu(dppb)**; Thus, we measured diffusion-ordered NMR spectroscopy (DOSY-NMR) in order to clarify whether **Cu(dppb)** is dimerized even in solutions. The obtained DOSY-NMR spectra of **Cu(dppb)**, **Cu(dppp)**, **Cu(dppe)**, **Cu(Xantphos)**, and [Cu(dmpp)_2_](PF_6_) measured in CD_2_Cl_2_ solutions are illustrated in Figure S7. The diffusion coefficients of **Cu(dppb)**, **Cu(dppp)**, **Cu(dppe)**, **Cu(Xantphos)**, and [Cu(dmpp)_2_](PF_6_) in the solutions were determined to be 7.5 × 10^−10^, 7.9 × 10^−10^, 8.0 × 10^−10^, 7.0 × 10^−10^ and 8.4 × 10^−10^ [m^2^/s], respectively. These obtained values were quite similar though they gradually decreased with increasing the molecular weight calculated as mononuclear complexes. Hence, the molecular sizes of the Cu(I) complexes might be almost the same in solutions. Since **Cu(dppp)** and **Cu(dppe)** were reported as mononuclear complexes in the solid state, **Cu(dppb)** in the reaction solutions is regarded as a mononuclear complex in this paper and the dimerization should not be the main reason for the high TOF of the system using **Cu(dppb)**.

#### Absorption Abilities of the Cu(I) Complexes

**Figure 4:**
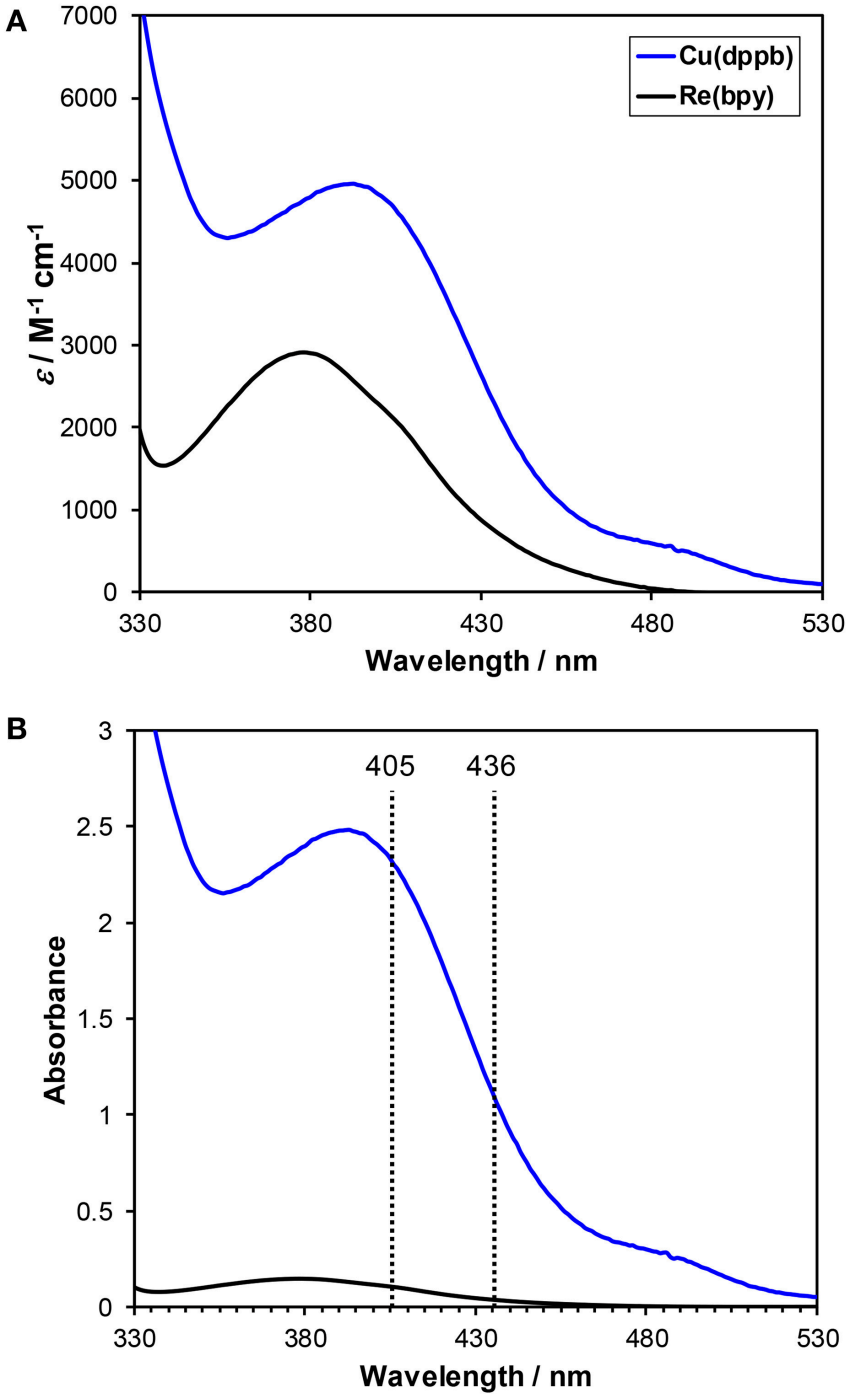
**(A)** UV-Vis absorption spectra of **Cu(dppb)** (blue) and **Re(bpy)** (black). The solvent was DMA-TEOA mixed solutions (4:1 v/v). **(B)** Normalized spectra by the concentration of the reaction condition for the photocatalytic reactions ([**Cu(dppb)**] = 0.5 mM, [**Re(bpy)**] = 0.05 mM).

Figure 4A shows the UV-vis absorption spectra of **Cu(dppb)** and **Re(bpy)** in a DMA-TEOA mixed solution. Both complexes showed MLCT absorption bands at similar region with similar molar extinction coefficients. However, in the reaction condition, **Cu(dppb)** can absorb visible light more efficiently than **Re(bpy)** because the used concentration of **Cu(dppb)** was 10-times higher than that of **Re(bpy)** (Figure 4B). The light source was a high-pressure mercury lamp equipped with a UV-cut filter (>370 nm); Therefore, the main wavelength of the excitation light should be 405 and 436 nm. At 405 and 436 nm, more than 96% of absorbed photons should excite **Cu(dppb)** [Abs_405_(**Cu(dppb)**): Abs_405_(**Re(bpy)**) = 2.3: 0.10, Abs_436_(**Cu(dppb)**): Abs_436_(**Re(bpy)**) = 1.1: 0.03]. This means **Cu(dppb)** was selectively excited by the excitation light under the reaction condition.

**Figure 5:**
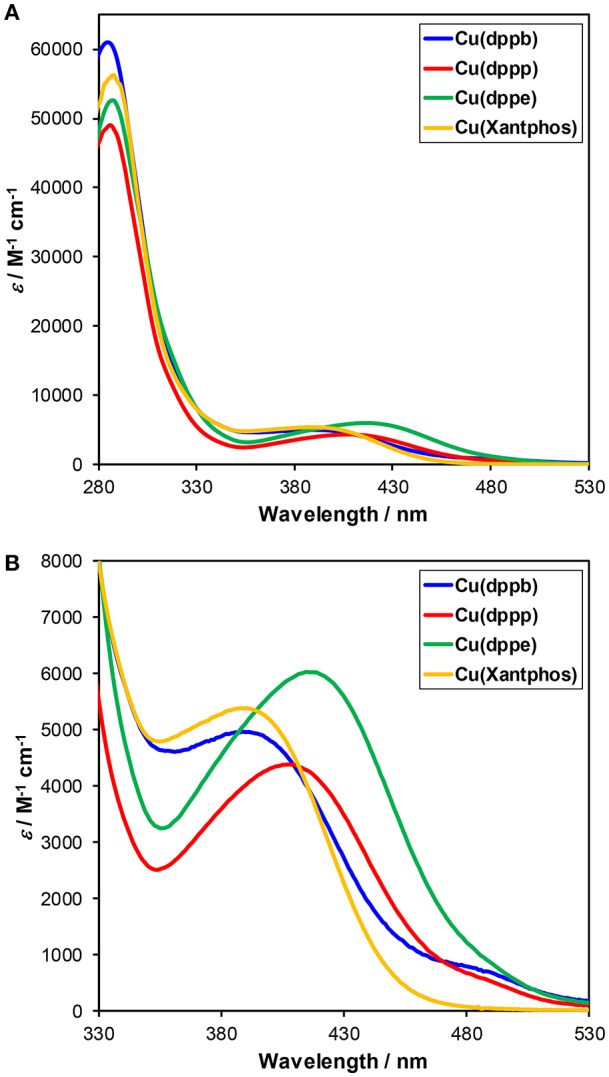
**(A)** UV-Vis absorption spectra of Cu(I) complexes, i.e., **Cu(dppb)** (blue), **Cu(dppp)** (red), **Cu(dppb)** (green), and **Cu(Xantphos)** (orange). The solvent was DMA-TEOA mixed solutions (4:1 v/v). **(B)** Enlarged spectra at λ_abs_ = 330–530 nm.

The UV-Vis absorption spectra of the series of the Cu(I) complexes in DMA-TEOA mixed solutions are illustrated in Figure 5. All of the Cu(I) complexes showed MLCT absorption bands at around 350–450 nm. When comparing **Cu(dppb)**, **Cu(dppp)**, and **Cu(dppe)**, the absorption maxima of the MLCT absorption bands were gradually blue-shifted with increasing the length of the carbon chains [λ_abs_(**Cu(dppe)**) = 417 nm, λ_abs_(**Cu(dppp)**) = 410 nm, λ_abs_(**Cu(dppb)**) = 391 nm]. Similar blue-shift of the absorption maxima was also observed in the reported system using dichloromethane solutions (Tsubomura et al., 2015). It is reported that the shift was strongly related to the bite angles of the diphosphine ligands, which were determined using the single crystal X-ray diffraction, i.e., the absorption energy became larger as bite angle became larger. The wavelength of the absorption maximum of **Cu(Xantphos)** was the shortest (388 nm); thus, the bite angle of Xantphos in **Cu(Xantphos)** might be larger than those in the other complexes. It should be noted that the absorbance at the irradiation wavelength (405, 436 nm) are relatively high (Abs > 0.86). Therefore, the total of the absorbed photon number of each reaction solution for the photocatalyses should be almost identical regardless of the structure of the diphosphine ligands, and TOFs should reflect the relative quantum yields for the photocatalytic CO_2_ reduction.

#### Photophysical and Photochemical Properties

In order to investigate the efficiencies of the initial steps of the photosensitizing reactions, we tried to measure the photophysical properties of Cu(I) complexes in the main solvent for the photocatalyses, i.e., DMA-TEOA mixed solutions. Interestingly, however, the Cu(I) complexes showed quite low luminescence properties in DMA-TEOA mixed solutions, even though the photocatalytic reactions proceeded efficiently in them. In particular, the emission quantum yield (Φ_em_) of **Cu(dppb)** in the DMA-TEOA solutions was <0.01% and emission lifetime (τ_em_) was shorter than 30 ns in contrast to those in other solvents (Φ_em_ in CH_2_Cl_2_ = 16%, τ_em_ in CH_2_Cl_2_ = 10 μs). The reason for the quite low luminescence properties of **Cu(dppb)** in DMA-TEOA solutions is now under investigation, but it might be caused by an efficient exciplex-quenching by DMA molecules (McMillin and McNett, 1998; Kuang et al., 2002). The detection of the luminescence from the DMA-TEOA solutions containing **Cu(dppb)** was difficult; therefore, we decided to compare the photophysical and photochemical properties of the series of Cu(I) complexes in the other solvents for the catalyses, i.e., a mixture of MeCN-TEOA. The emission properties of the Cu(I) complexes in a mixture of MeCN-TEOA (4:1 v/v) are summarized in Table 2 and the emission spectra are shown in Figure S8. All of the complexes showed weak emission intensity and short emission lifetimes in a mixture of MeCN-TEOA compared with those in dichloromethane likely due to the exciplex-quenching by the MeCN molecules. In fact, the emission intensity of **Cu(dppb)** in MeCN solutions was almost identical to that in MeCN-TEOA solutions. Hence, the low emission intensity observed should not be derived from the quenching by TEOA (Figure S9). In accordance with the blue-shift of the MLCT absorption bands in the absorption spectra, the wavelength of the emission maxima of the three complexes, i.e., **Cu(dppb)**, **Cu(dppp)**, and **Cu(dppe)**, gradually decreased as the number of the methylene chains in the diphosphine ligands increased. The emission lifetimes became longer with increasing the length of the carbon chains. This is likely because the steric hindrance of the phenyl groups on the phosphorous atoms became larger with increasing the bite-angle of the diphosphine ligands, which resulted in inhibiting the attack of the solvent molecules in the excited state and decreasing non-radiative decay constants. **Cu(Xantphos)** showed the shortest wavelength of the emission maximum and the longest emission lifetime.

**Table 2:** Photophysical properties of Cu(I) complexes in a mixed solution of MeCN-TEOA (4:1 v/v)^a^.

**Complex**	**$\lambda_{{\text{abs}}^{b}}$/nm (ε/M^**−1**^s^**−1**^)**	**λ_em_/nm**	**Φ_em_**	**${\text{τ}}_{{\text{em}}^{\text{f}}}\text{/μs}$**
**Cu(dppb)**	387 (4,700)	605^c^	0.003^c^	0.24
	388 (5,000)^g^	585^g^	0.16^g^	10^g^
**Cu(dppp)**	400 (4,000)	608^d^	<0.001^d^	0.015
**Cu(dppe)**	411 (5,200)	608^e^	<0.001^e^	0.004
**Cu(Xantphos)**	388 (5,200)	577^c^	0.007^c^	0.36

a *Measured in a mixture of MeCN-TEOA*.

b *The molar extinction normalized by the number of the Cu centers. The UV-Vis absorption spectra are shown in Figure S8*.

c *Excitation wavelength: 390 nm*.

d *Excitation wavelength: 400 nm*.

e *Excitation wavelength: 410 nm*.

f *Excitation wavelength: 337 nm*.

g *The repoted values measured in dichloromethane (Tsubomura et al., 2015)*.

**Figure 6:**
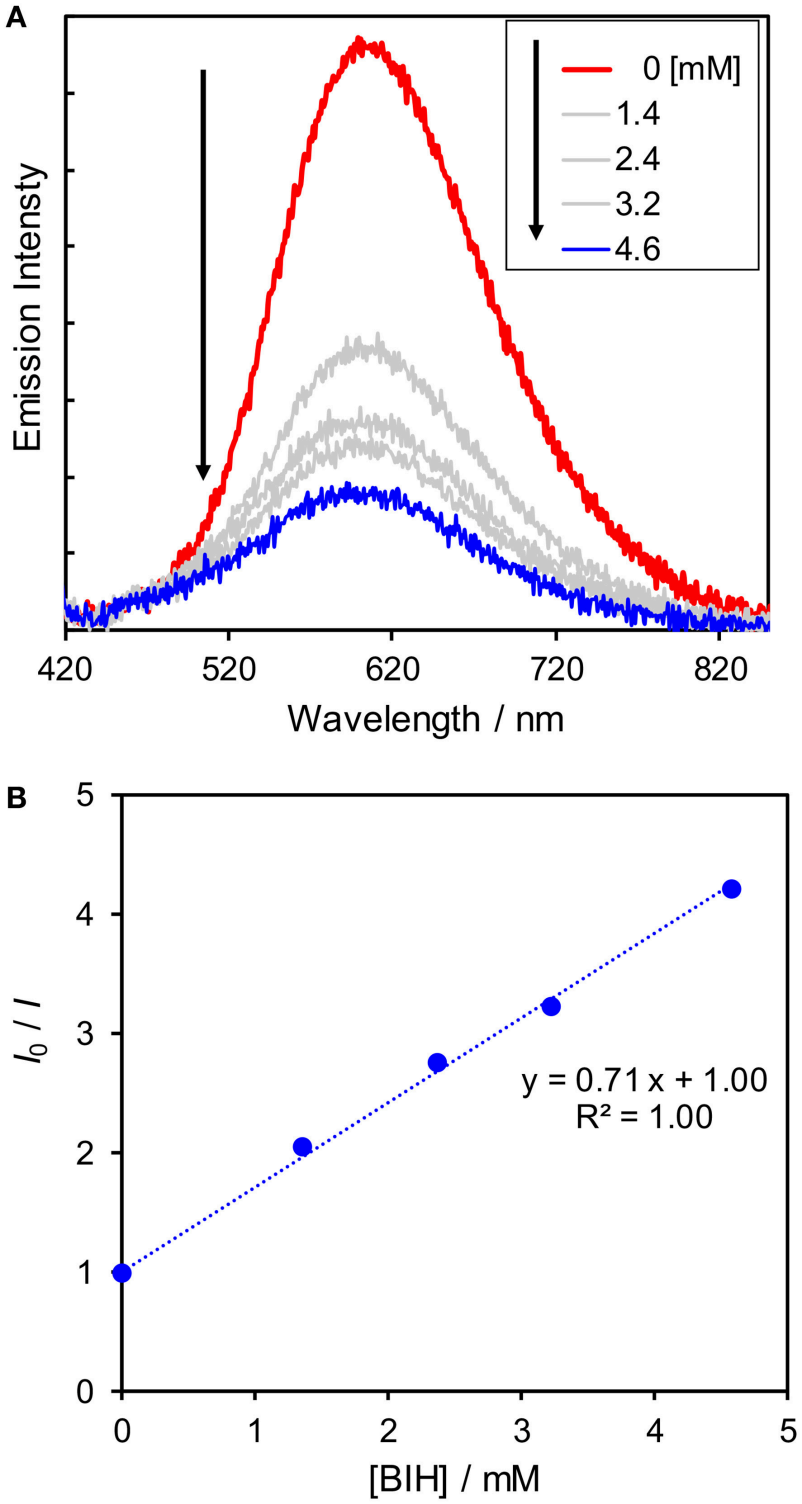
**(A)** Emission spectra (excitation 410 nm) of **Cu(dppb)** in MeCN-TEOA under an Ar atmosphere in the presence of various amounts of BIH and **(B)** the Stern-Volmer plot using emission intensity (*I*) and the emission intensity without any BIH (*I*_0_).

Since the reactions between the excited state of the Cu(I) complexes and BIH compete with the radiative and non-radiative deactivation processes, the shorter lifetime might lower the efficiencies of the quenching by BIH. Thus, we conducted quenching experiments using BIH to check whether the excited state of the Cu(I) complexes can be quenched by BIH. We measured emission intensity in the presence of various amounts of BIH. As shown in Figure 6A, the emission intensities became smaller with increasing the concentrations of BIH, indicating the excited state of **Cu(dppb)** was efficiently quenched by BIH. The Stern–Volmer analysis showed linear plots with an intercept at 1 (Figure 6B). From the slope of the fitting curve (*K*_SV_ = *k*_q_τ_em_ = 0.71 mM^−1^) and the emission lifetime τ_em_ = 240 ns, the quenching rate constant (*k*_q_) was determined to be 3.0 × 10^9^ M^−1^ s^−1^. From these values, the quenching fraction (η_q_) is estimated to be larger than 98% under the reaction condition (Equation 2) and the excited state of **Cu(dppb)** is expected to be quenched by BIH almost quantitatively, even though the emission lifetime is not so long. Moreover, in the presence of **Re(bpy)**, the emission intensity was almost identical to that in the absence of **Re(bpy)** (Figure S10); therefore, oxidative quenching, i.e., the electron transfer from the excited state of **Cu(dppb)** to **Re(bpy)** should be negligible. These results suggest that the photosensitizing reaction by **Cu(dppb)** mainly proceeds via the reductive quenching by BIH.

(2) $$\begin{array}{r} \eta_{\text{q}}\;=\;\left[\text{BIH}\right]\times {\text{K}}_{\text{SV}}/\left(1+\left[\text{BIH}\right]\times {\text{K}}_{\text{SV}}\right) \end{array}$$

In the case using the other Cu(I) complexes, the emission intensity also decreased drastically by adding BIH, and emission from the Cu(I) complexes could not be detected in the presence of 0.1 M BIH. The Stern–Volmer analysis using the other Cu(I) complexes not only in a mixture of MeCN-TEOA but also in a mixture of DMA-TEOA could also be conducted owing to the larger emission intensities and the longer emission lifetimes in DMA-TEOA mixed solutions than those of **Cu(dppb)**. The Stern–Volmer analysis showed linear plots with an intercept at 1 in all cases (Figure S11). From the slope of the fitting curve, the quenching fractions of **Cu(dppp)**, **Cu(dppe)**, and **Cu(Xantphos)** were estimated to be larger than 99, 98, and 99%, respectively. These results clearly indicate that all of the Cu(I) complexes used in this study have relatively strong oxidizing power in the excited state and the efficiencies of the initial stage of the photosensitizing reaction, i.e., reductive quenching, should be almostidentical regardless of the structure of the diphosphine ligands.

#### Spectral Changes During Irradiation

**Figure 7:**
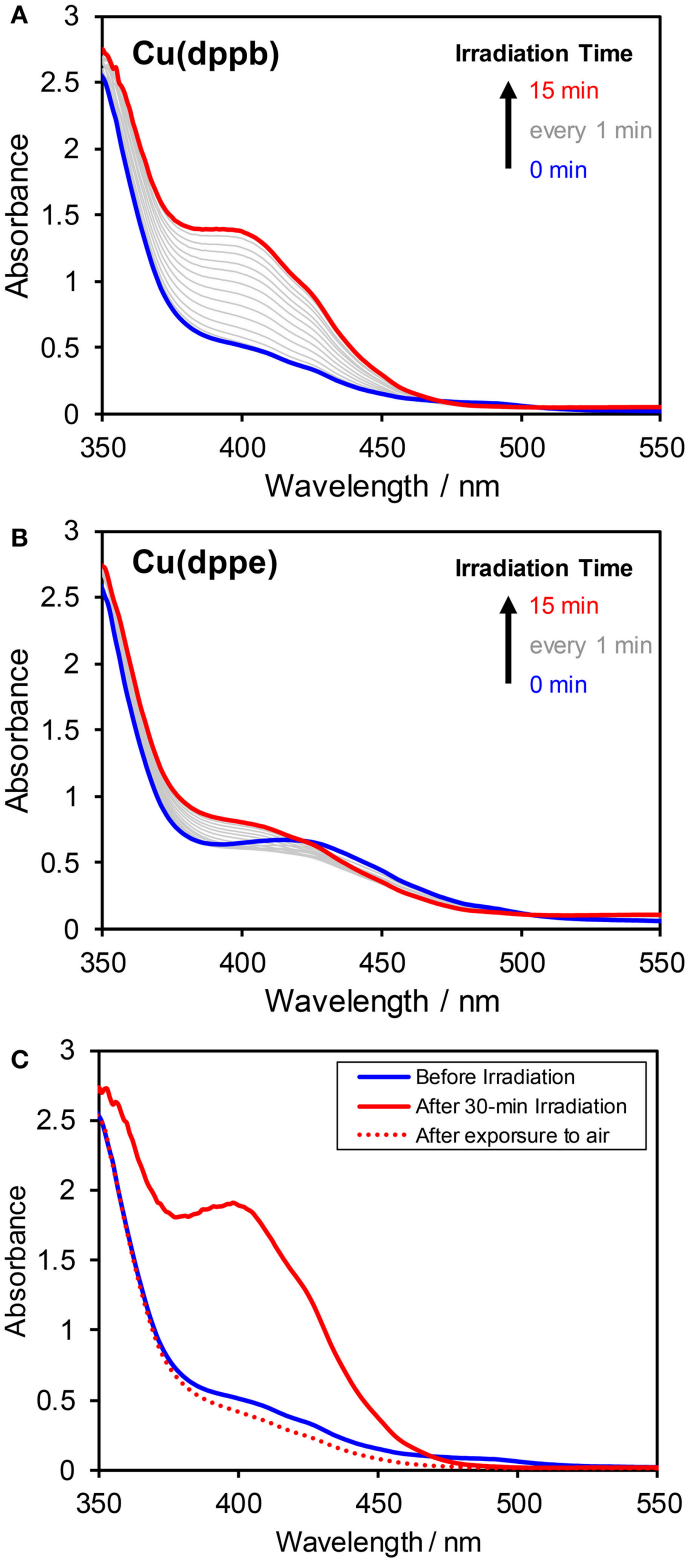
Absorption spectral changes of DMA–TEOA (4:1 v/v) solutions containing BIH (0.1 M) and 0.1 mM of **(A) Cu(dppb)** or **(B) Cu(dppe)** under an argon atmosphere. The solution was irradiated with 430-nm monochromic light. **(C)** Absorption spectral changes of the reaction solution of **Cu(dppb)** measured before irradiation (blue solid line), just after 30-min irradiation (red solid line), and after irradiation and subsequent exposure to an ambient air (red dotted line).

As mentioned above, the excited state of the Cu(I) complexes was efficiently quenched by BIH. To clarify the produced species of the quenching, UV-vis absorption spectral changes during photo-irradiation of a DMA-TEOA mixed solution (4:1 v/v) containing **Cu(dppb)** (0.1 mM) and BIH (0.1 M) under Ar were measured (Figure 7A). After an induction period over a few minutes, a new absorption bands at around 350–450 nm was observed. The new band increased continuously and linearly over 30-min irradiation. After irradiation, the solution was exposed to an ambient air. The solution color was immediately changed from deep-yellow to light-yellow. In the absorption spectrum measured after exposure to air, the new absorption band was completely disappeared and the original spectrum-shape was recovered (Figure 7C). These results clearly indicate that the quenching of the excited state of **Cu(dppb)** is a reductive quenching process, which gives one-electron reduced species (OERS) of **Cu(dppb)**. In contrast, when using **Cu(dppe)** the observed spectral changes were much smaller than **Cu(dppb)** though the shape of the new absorption band was similar to those of **Cu(dppb)** (Figure 7B). This difference should be derived from the difference of efficiencies for production of OERS between **Cu(dppb)** and **Cu(dppe)**. **Cu(Xantphos)** also showed rapid accumulation of OERS under the same condition, and **Cu(dppp)**, in contrast, showed slow accumulation as well as **Cu(dppe)** (Figure S12). Though the determination of the exact rate of production of OERS requires a spectrum obtained by electrochemical spectroscopy technics, such big difference in spectral changes and similarity of the shapes of the new absorption bands should suggest that the efficiency for production of OERS of **Cu(dppb)** and **Cu(Xantphos)** should be much larger than those of **Cu(dppp)** and **Cu(dppe)**. The tendency of the degree of the spectral changes corresponded reasonably well with that of TOF described above.

#### Electrochemical Properties

**Figure 8:**
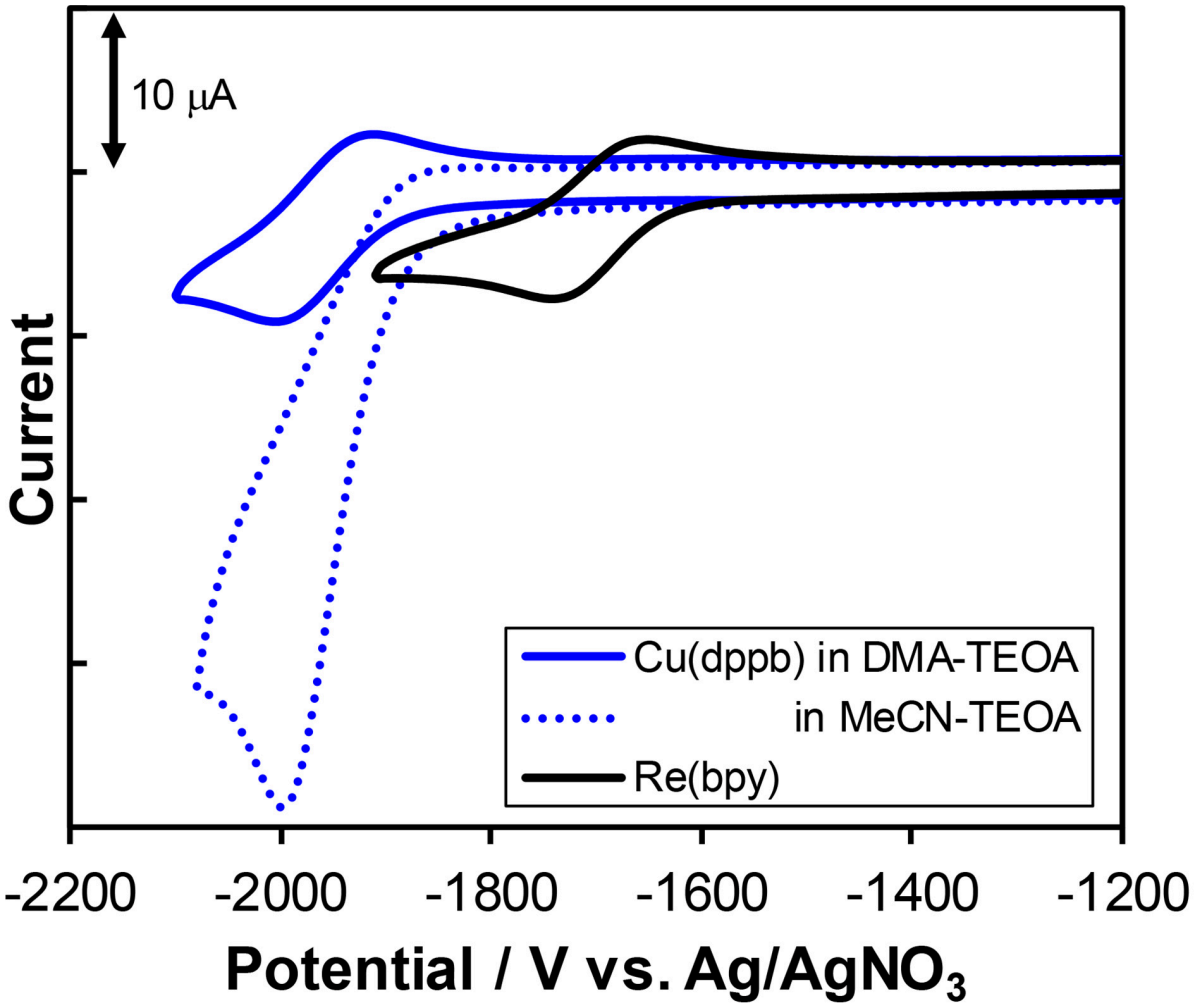
Cyclic voltammograms of **Cu(dppb)** (blue solid line) and **Re(bpy)** (black solid line) a mixture of DMA and TEOA (4:1 v/v) containing Et_4_NPF_6_ (0.1 M) as a supporting electrolyte with a Ag/AgNO_3_ (0.01M) reference electrode. The voltammogram of **Cu(dppb)** measured in a mixture of MeCN and TEOA (4:1 v/v) is also illustrated as a blue dotted line.

Figure 8 shows the cyclic voltammograms of **Cu(dppb)** and **Re(bpy)** measured in DMA-TEOA solutions and their redox potentials are summarized in Table 3. Both **Cu(dppb)** and **Re(bpy)** exhibited a quasi-reversible reduction wave as a first reduction wave at −1.96 and −1.70 V (vs. Ag/AgNO_3_), respectively. These waves are attributable to the reduction of the diimine ligands. The reduction potential of **Cu(dppb)** is more negative than that of **Re(bpy)**; thus, OERS of **Cu(dppb)** has relatively strong reducing power and the electron transfer process from OERS of **Cu(dppb)** to **Re(bpy)** occurs exothermically when using **Cu(dppb)** as a photosensitizer and **Re(bpy)** as a catalyst. Interestingly, when using an MeCN-TEOA solution, the first reduction wave of **Cu(dppb)** became irreversible, though the peak potential was almost identical to that in the DMA-TEOA solution. This means OERS of **Cu(dppb)** is not stable in an MeCN-TEOA solution and might be one of the reasons for the low TON_CO_ in the case using MeCN-TEOA solutions. Even though the oxidation potentials of the complexes could not be observed due to the potential window narrowed by oxidation of TEOA, the irreversible oxidation wave was observed at +0.80 V when using a DMA solution instead of the DMA-TEOA solutions. The first reduction potential observed in the DMA solution was almost identical to that in the presence of TEOA (−1.91 V). **Cu(dppb)** showed more negative reduction potential than Ru(II) tris-diimine complexes, which are typical redox-photosensitizers in the photocatalytic systems for CO_2_ reduction. On the other hand, the oxidation potential was similar to those of the Ru(II) complexes; (Yamazaki et al., 2015) thus, **Cu(dppb)** should have both high reducing power in the reduced state and relatively high oxidizing power in the excited state.

**Table 3:** Electrochemical properties of the metal complexes in DMA-TEOA^a^.

**Complex**	***E***_**1/2**_**/V vs. Ag/AgNO**_**3**_ **(***ΔE***/mV)**	
	**M(N^**∧**^N/N^**∧**^N^**−**^) (M = Cu or Re)**	**Cu^**I/II**^**
**Cu(dppb)**	−1.96 (93)	—
	−1.91 (76)^b^	+0.80^b, c^
**Cu(dppp)**	−1.95 (84)	—
**Cu(dppe)**	−1.93 (78)	—
**Cu(Xantphos)**	−1.96 (79)	—
**Re(bpy)**	−1.70 (78)	—

a *Measured in a DMA-TEOA mixed solution containing the complex (0.5 mM) and Et_4_NPF_6_ (0.1 M) with a scan rate of 100 mV·s^−1^ under an Ar atmosphere*.

b *A DMA solution was used instead*.

c *Peak potential*.

The other Cu(I) complexes also showed reversible or quasi-reversible waves in the cyclic voltammograms measured in DMA-TEOA solutions (Figure S13). The reduction potentials were almost identical (−1.93 to −1.96 V); the reduction potentials were not strongly affected by the structure of the diphosphine ligands. These results are reasonable because it is well known that the lowest-unoccupied molecular orbitals (LUMO) of the Cu(I) complexes are mainly derived from the π^*^-orbitals of diimine ligands and the series of Cu(I) complexes have the same diimine ligands. Therefore, all of the Cu(I) complexes should have high reducing power in the reduced state enough to smoothly trigger the electron transfer from OERS of the Cu(I) complexes to **Re(bpy)**.

#### Expected Reaction Mechanism and Relationship Between the Molecular Structure and the Photosensitizing Abilities

**Figure 9:**
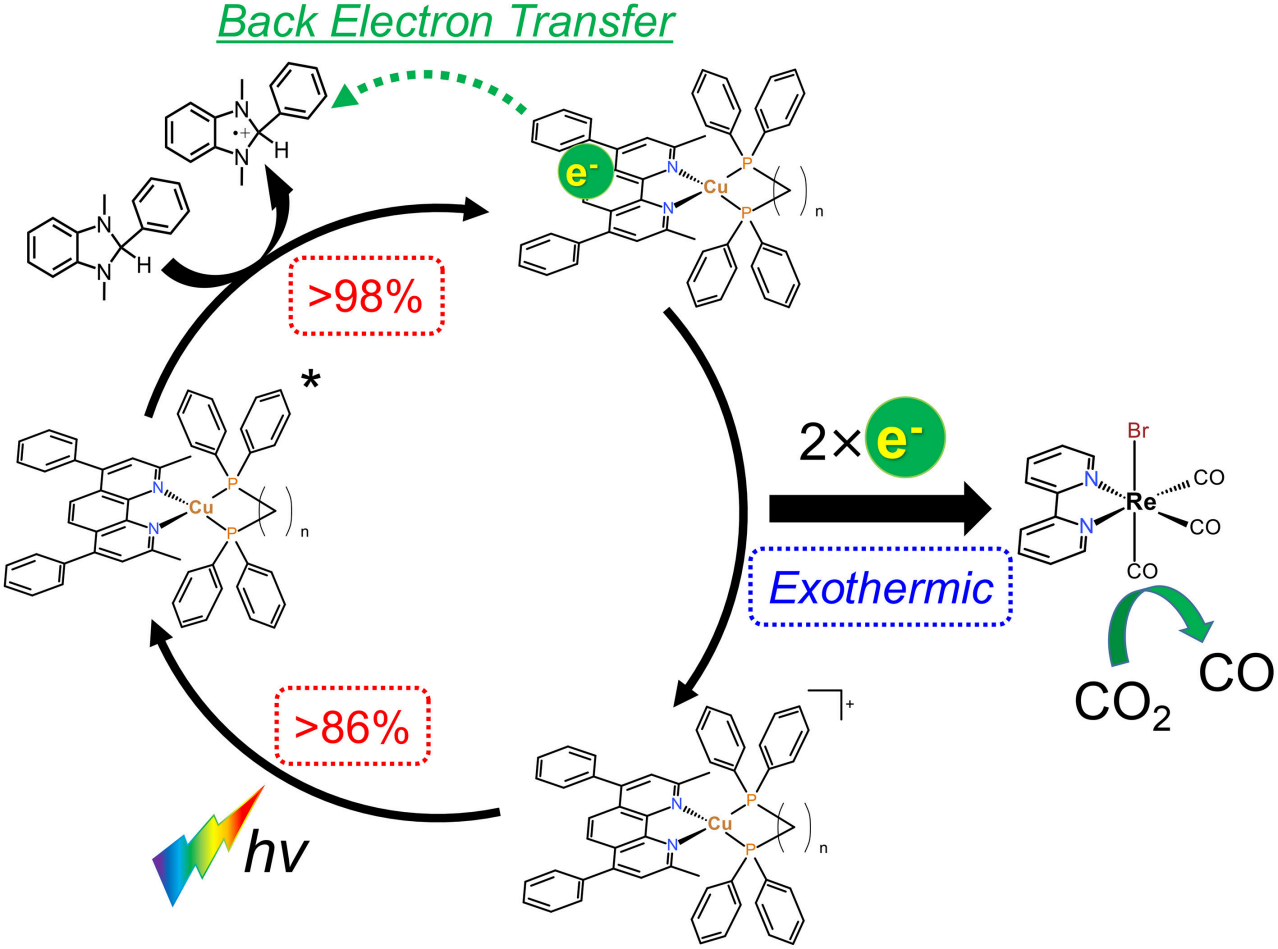
Expected reaction mechanism of the CO_2_ reduction using the Cu(I) complexes, **Re(bpy)** and BIH.

From the results in the previous sections, the expected reaction mechanism of the photocatalytic systems in this study can be summarized as follows (Figure 9): (1) almost all of the irradiated photons are absorbed by the Cu(I) complexes; (2) the excited states of the Cu(I) complexes are reductively quenched by BIH to give OERS with high efficiencies; (3) the electron transfer from the OERS of the Cu(I) complexes to **Re(bpy)** proceeds exothermically; (4) CO_2_ reduction takes place on **Re(bpy)** using the obtained electrons.

**Figure 10:**
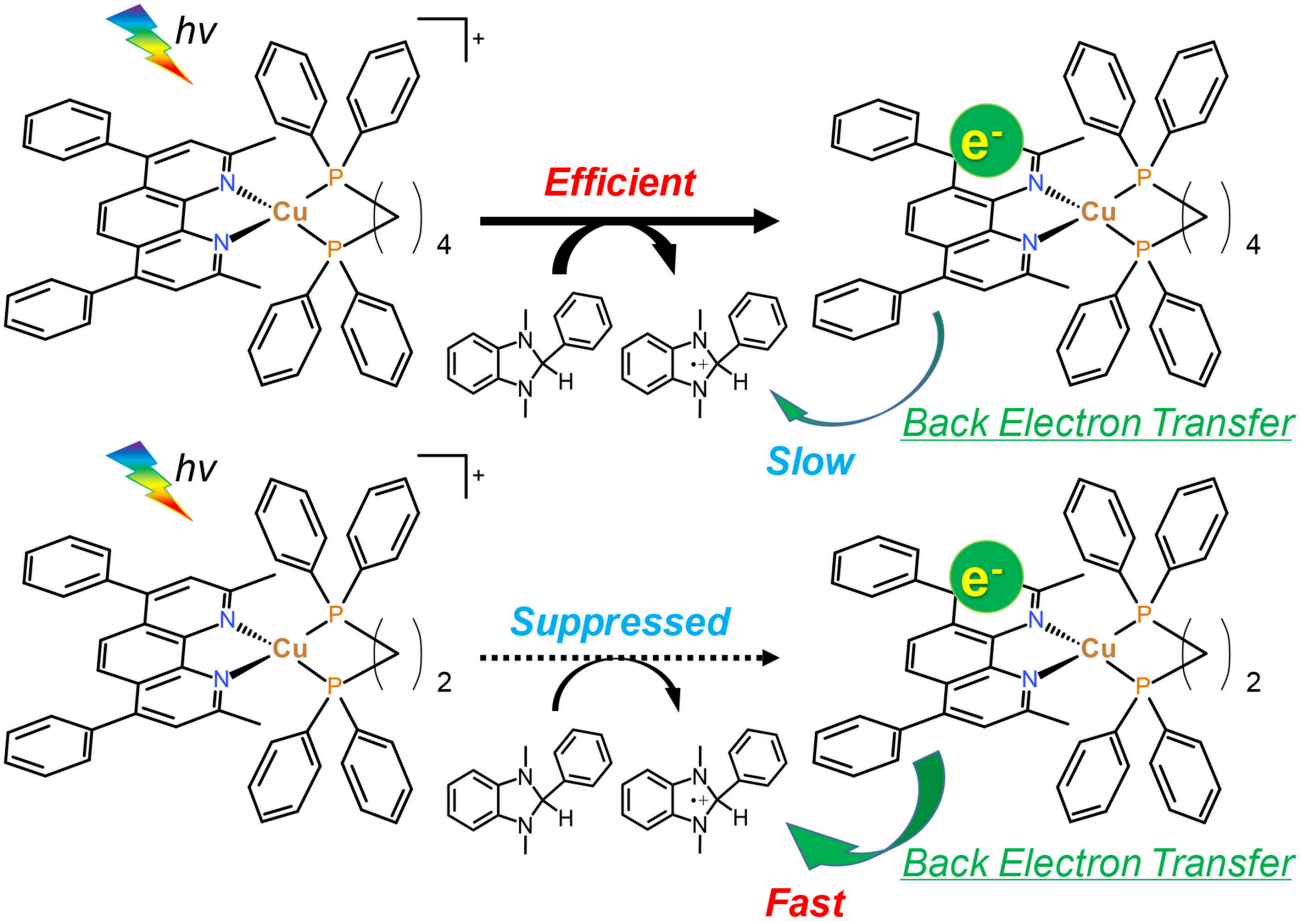
The one-electron reduction processes of **Cu(dppb)** and **Cu(dppe)**.

As described in section Photocatalytic CO_2_ reduction using Cu(I) complexes as photosensitizers, TOFs were drastically changed by changing the structure of the diphosphine ligands. In contrast, all of the Cu(I) complexes showed quite similar absorbance, quenching fractions and reduction potentials; therefore, we could not observe a big difference in the efficiency of each step illustrated in Figure 9. However, only the efficiencies of the production of OERS strongly depended on the molecular structure even though the preceding process, i.e., the reductive quenching, proceeded almost quantitatively in all cases. It is reported that the efficiencies for the production of OERS strongly affected not only by the quenching fractions but also by the rate of the back electron transfer processes, i.e., the electron transfer from the OERS of the Cu(I) complexes to the one-electron oxidized species of BIH (BIH^·+^) produced by the reductive quenching processes (Tamaki et al., 2013a). This charge-recombination process forms the ground states of the Cu(I) complexes and BIH, resulting in wasting absorbed photons. The low efficiencies for the production of OERS in the case using **Cu(dppe)** should indicate that the back electron transfer proceeded more rapidly than the case using **Cu(dppb)** (Figure 10). The reason for the great difference in the rate of the back electron transfer is unclear at the moment, but the difference in the bite angles of the diphosphine ligands might be an important factor. The orientation of the four phenyl groups on the two phosphorous atoms is affected by changing the bite angles of the diphosphine ligands. Along with this, the distance between the phenyl groups on the phosphine ligands and the dmpp ligands will decrease by increasing the bite angles. The electrons obtained photochemically should mainly localize on the π^*^ orbital in the dmpp ligands; thus, the back electron transfer process should proceed via the attack of BIH^·+^ to the π^*^ orbital. If the phenyl groups exist nearby the dmpp ligands and cause the steric hindrance, the access of BIH^·+^ will be suppressed. As a result, the large P-Cu-P angles might suppress the charge-recombination and enhanced the efficiencies for OERS production. In fact, not only the photophysical properties but also TOFs have good correlation with the reported bite angles determined using the single crystal X-ray diffraction (Table 4); (Tsubomura et al., 2015; Heberle et al., 2017) in other words, not only long emission lifetimes but also high photosensitizing abilities will be obtained by increasing the bite angles of the diphosphine ligands likely due to the steric hindrance of the phenyl groups to suppress the approach of the solvent molecules to the Cu(I) center and BIH^·+^ to the dmpp ligands.

**Table 4:** Relationship between P-Cu-P angles in the solid state of the Cu(I) complexes and photosensitizing abilities^a^.

**Complex**	**P-Cu-P angles^**a**^/^**°**^**	**TOF/min^**−1**^**	***λabs^d^/**nm*****	**$\lambda abs^{d}/nm$**	**${}^{\tau }em^{e/\mu s}$**
**Cu(dppb)**	120^b^	65	391	0.24
**Cu(dppp)**	105^b^	27	410	0.015
**Cu(dppe)**	91^b^	7.3	417	0.004
**Cu(Xantphos)**	113^c^	72	388	0.36

a *The angles are the reported values determined by single crystal X-ray diffraction studies. TOF was determined by the slope of the fitting curves over 5 min (Figure S2)*.

b *The values from the reported literature (Tsubomura et al., 2015)*.

c *The values from the reported literature (Heberle et al., 2017). The value in [Cu(2,9-dimethyl-1,10-phenanthroline)(Xantphos)]^+^ instead of **Cu(Xantphos)***.

d * Measured in a mixture of DMA-TEOA (4:1 v/v)*.

e * Measured in a mixture of MeCN-TEOA (4:1 v/v)*.

## Experiments

### General Procedure

NMR spectra were recorded on a JEOL ECA500 (500 MHz) spectrometer. The spectra were analyzed by Delta version 5. Transmission UV-vis (ultraviolet-visible light) absorption spectra were recorded on an Agilent 8453 spectrometer. Emission spectra were obtained in a solvent degassed by bubbling with argon. Emission spectra and emission lifetimes were recorded on a laboratory-made apparatus. The samples were excited by a monochromated xenon light source and the emission was analyzed by using a cooled CCD spectrometer. For the lifetime measurement, a nitrogen laser (USHO AN-200) was used to excite the samples, and the emission was detected by a monochromator equipped with a photomultiplier tube, and the signal was analyzed by using a digital oscilloscope. Emission quantum yields in solution were determined a combination of an integrating sphere, a monochromated xenon light source and a cooled CCD spectrometer.

### Electrochemical Measurement

Cyclic voltammograms were conducted using an electrochemical analyzer, EC-stat 100 (EC Frontier CO., Ltd), using an electrochemical cell equipped with working (glassy carbon, φ = 3 mm), auxiliary (platinum wire), and reference (Ag/AgNO_3_, 10 mM) electrodes. The solution containing a Cu(I) complex (0.5 mM) and Et_4_NPF_6_ (0.1 M) was bubbled with argon before the measurements.

### Materials

**Cu(dppb)**, **Cu(dppp)**, **Cu(dppe)**, **Re(bpy)**, and BIH were prepared according to reported methods with some modifications (Hawecker et al., 1986; Tamaki et al., 2013a; Tsubomura et al., 2015). **Cu(Xantphos)** were synthesized using dmpp instead of the corresponding diimine ligand according to the method for [Cu(2,2′-biquinoline)(Xantphos)](PF_6_) (McCullough et al., 2018). Other chemicals were used as purchased without further purification.

### Photocatalytic Reactions

The photocatalytic reactions were performed in a 15 mL test tube (i.d. = 11 mm) containing a 2 mL DMA–TEOA (4:1 v/v) solution of **Re(bpy)** (0.05 mM), BIH (0.1 M), and a Cu(I) complex (0.5 mM) after purging with CO_2_ for more than 20 min. The solution was irradiated using a laboratory-made merry-go-round irradiation apparatus at λ > 370 nm with a high-pressure mercury lamp (SEN LIGHT Co.) combined with a UV-cut filter (Lintec Commerce. Inc.). During irradiation, the tube was cooled with a thermostatic bath (20°C). The gaseous reaction products (CO and H_2_) were analyzed using a GC-TCD instrument (Shimadzu GC-8A).

### Measurement of the Absorption Spectrum of the OERS

A DMA–TEOA (4:1 v/v) solution (2 mL) containing the Cu(I) complex (0.1 mM) and BIH (0.1 M) in a 7 mL quartz cell (light-pass length: 1 cm) was purged with Ar for over 20 min. The solution was irradiated with 430-nm monochromic light derived from an LED lamp purchased from CELL System Co. UV-Vis absorption spectral changes during irradiation were recorded using a Shimadzu QYM-01 system.

### Measurement of Quantum Yields of Photocatalytic CO Formation

A DMA–TEOA (4:1 v/v) solution (2 mL) containing **Cu(dppb)** (0.5 mM), **Re(bpy)** (0.05 mM), and BIH (0.1 M) in a quartz cell (light-pass length: 1 cm, volume: 7 mL, light intensity: 1 × 10^−8^ einstein per s) was purged with CO_2_ for 30 min. The solution was irradiated at λ_ex_ = 430 nm derived from an LED lamp purchased from CELL System Co. UV-Vis absorption spectral changes during irradiation were recorded using a Shimadzu QYM-01 system. During irradiation, the cell was cooled at 25°C with a thermostatic bath. The gaseous reaction products (CO and H_2_) were analyzed using a GC-TCD instrument (Shimadzu GC-8A).

## Conclusion

We investigated in detail the effects of the structure of the diphosphine ligands on the photosensitizing abilities of heteroleptic diimine-diphosphine Cu(I) complexes using a series of Cu(I) complexes bearing dmpp ligands and 4 types of diphosphine ligands by conducting photocatalytic CO_2_ reduction using the Cu(I) complexes as photosensitizers. Turnover frequencies of the CO_2_ reduction drastically increased up to ~70 min^−1^ with increase of the P-Cu-P angles, even though all of the Cu(I) complexes showed similar photophysical, photochemical and electrochemical properties. In the case using **Cu(dppb)**, rapid accumulation of OERS was observed. On the contrary, when using **Cu(dppe)**, the apparent rate of the production of the OERS was quite slow. The low efficiency for the production of the OERS should be derived from the rapid charge-recombination with BIH^·+^. The large bite angles make the orientation of the phenyl groups on the phosphorous atoms toward the dmpp ligands, resulting in suppression of the attack by BIH^·+^ to the dmpp ligands. This might be one of the reasons for the high efficiencies of both the production of OERS and photocatalytic reactions when using the Cu(I) complexes connecting with the diphosphine ligands with the large bite angles.

## Author Contributions

YY and TT designed the study and YY wrote the initial draft of the manuscript. TO and TU investigated the photosensitizing abilities of the Cu(I) complexes. JI contributed to analyses of the structure of the Cu(I) complexes in solutions using NMR spectroscopy. SF measured the photophysical properties of the Cu(I) complexes in detail. The electrochemical properties of the Cu(I) complexes were checked by CT. All authors approved the final version of the manuscript, and agree to be accountable for all aspects of the work.

### Conflict of Interest Statement

The authors declare that the research was conducted in the absence of any commercial or financial relationships that could be construed as a potential conflict of interest.
