# Supplementary material for: Photocatalytic CO2 Reduction Using Various Heteroleptic Diimine-Diphosphine Cu(I) Complexes as Photosensitizers
Source: Front Chem. 2019 Apr 30;7:288. doi: 10.3389/fchem.2019.00288 (PMC6502988; doi:10.3389/fchem.2019.00288)
Supplement: Supplementary file 1 [file Data_Sheet_1.PDF]

Supplementary Material

Photocatalytic CO_2 Reduction Using Various Heteroleptic Diimine-Diphosphine Cu(I) Complexes as Photosensitizers

Yasuomi Yamazaki, Takayuki Onoda, Jun Ishikawa, Shota Furukawa, Chinatsu Tanaka, Tomoya Utsugi, and Taro Tsubomura*

*** Correspondence:** Dr. Taro Tsubomura
E-mail: tsubomura@st.seikei.ac.jp; Fax: +81-422-37-3871; Tel: +81-422-37-3752

1 Supplementary Figures

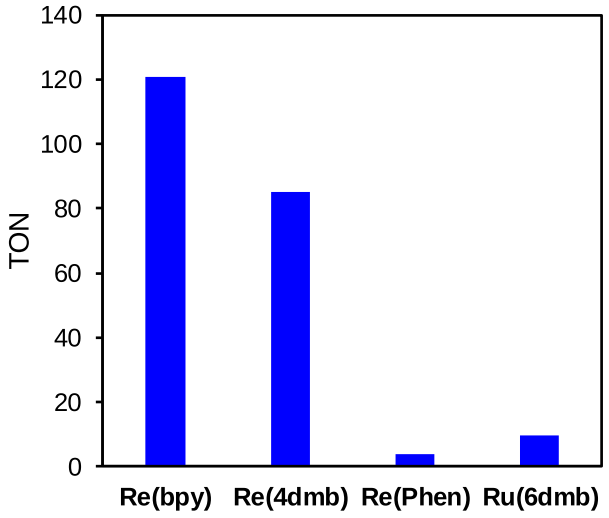

Figure S1. Photocatalytic production of CO over 1-h irradiation using **Re(bpy)**, Re(4,4'-dimethyl-2,2'-bipyridine)$(CO)_3$Br (**Re(4dmb)**), Re(1,10-phenanthroline)$(CO)_3$Br (**Re(Phen)**), or Ru(6,6'-dimethyl-2,2'-bipiridine)$(CO)_2Cl_2$ (**Ru(6dmb)**): CO_2-saturated MeCN-TEOA (4:1 v/v) solutions containing **Cu(dppb)** (0.1 mM), a Re(I) complex (0.05 mM), and BIH (0.1M) were irradiated using a high-pressure mercury-lamp equipped with a UV-cut filter (>370 nm).

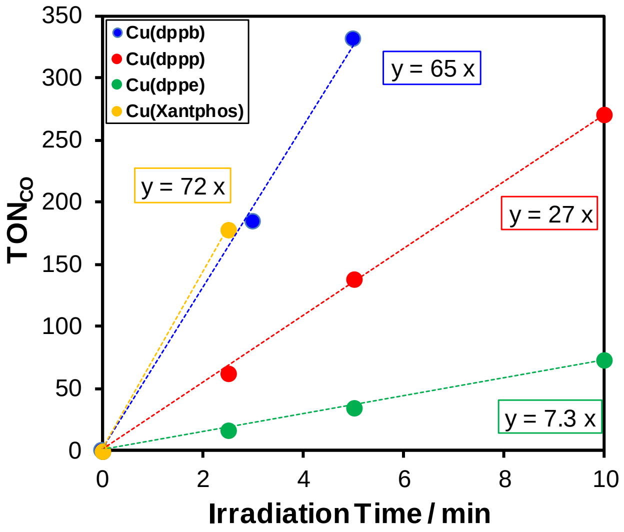

Figure S2. Time courses of the TON of CO formation in the initial stage of photocatalytic reactions (< 10 min, λ_{ex} > 370 nm) using a mixture of DMA and TEOA (4:1 v/v) containing 0.05 mM **Re(bpy)**, 0.1 M BIH, and Cu(I) complexes (blue: **Cu(dppb)**, red: **Cu(dppp)**, green: **Cu(dppe)**, orange: **Cu(Xantphos)**). The concentration of each Cu(I) complex was 0.5 mM. TOF was determined from the slopes of the fitting curves. In the case of **Cu(Xantphos)**, the fitting curve was drawn with the plot recorded within 2.5 min because decrease of the reaction rate was observed after 5-min irradiation.

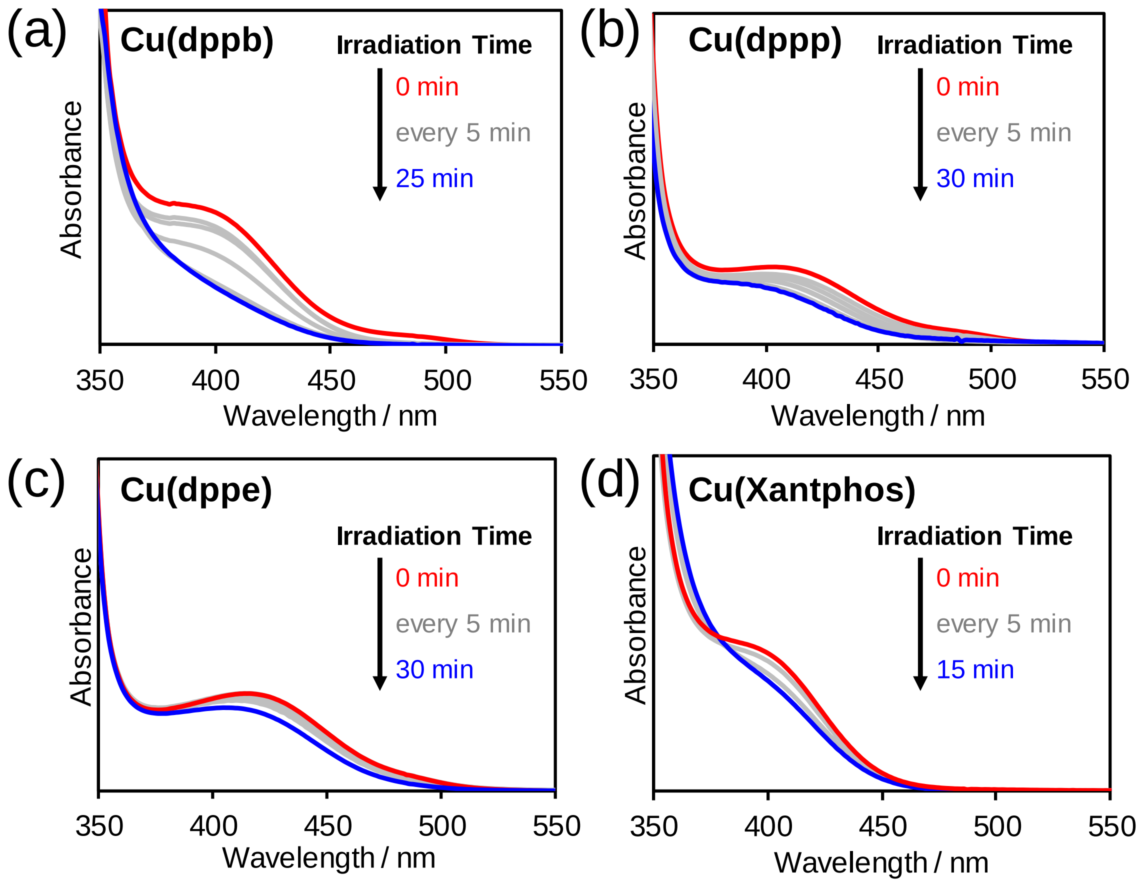

Figure S3. Absorption spectral changes of the reaction solutions containing (a)**Cu(dppb)**, (b)**Cu(dppp)**, (c)**Cu(dppe)** or (d)**Cu(Xantphos)** after the photocatalytic CO_2 reduction.

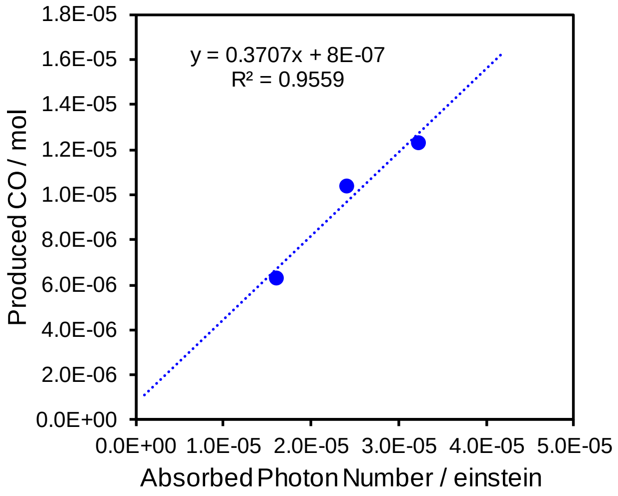

Figure S4. The relationship between the absorbed photon numbers and the amount of the produced CO using a mixture of DMA and TEOA (4:1 v/v) containing 0.5 mM **Cu(dppb)**, 0.05 mM **Re(bpy)** and 0.1 M BIH with 430-nm monochromic LED-light. Φ_{CO} was determined from the slopes of the fitting curves. The light intensity was 1×10^{-8} einstein/s.

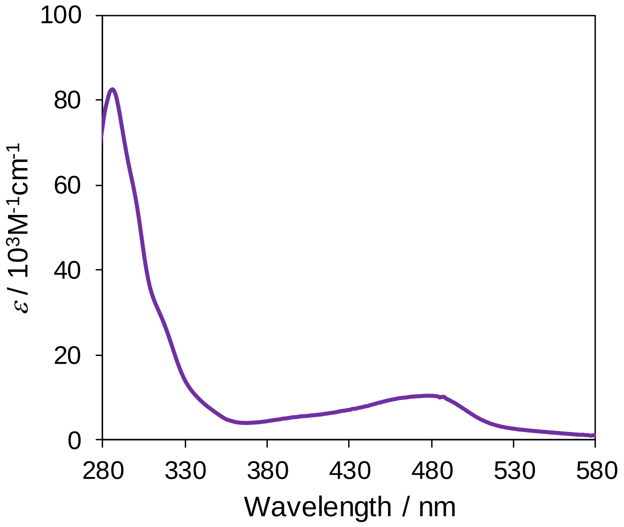

Figure S5. UV-vis absorption spectra of $[Cu(dmpp)_2](PF_6)$. The solvent was a mixture of DMA-TEOA (4:1 v/v).

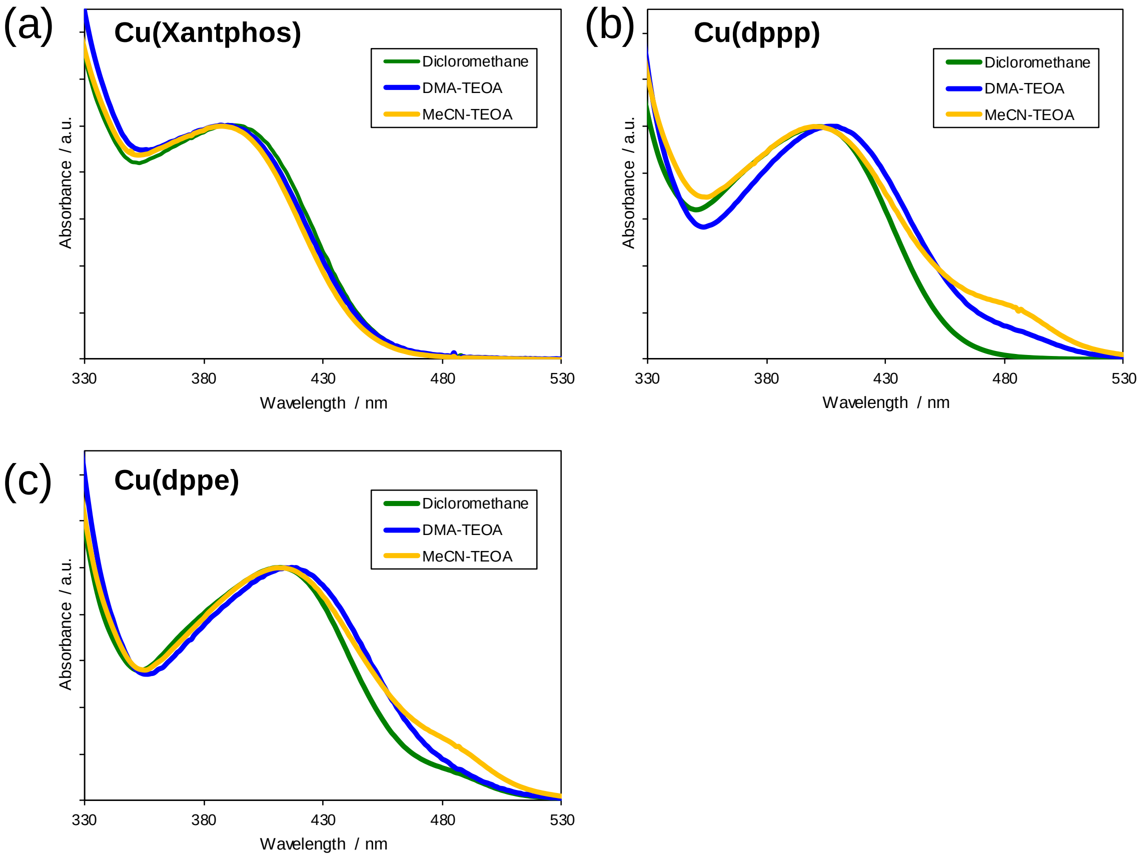

Figure S6. UV-Vis absorption spectra of (a) **Cu(Xantphos)**, (b) **Cu(dppp)** and (c) **Cu(dppe)** in various solutions: dichloromethane (green), a mixture of DMA-TEOA (4:1 v/v, blue), and a mixture of MeCN-TEOA (4:1 v/v, orange).

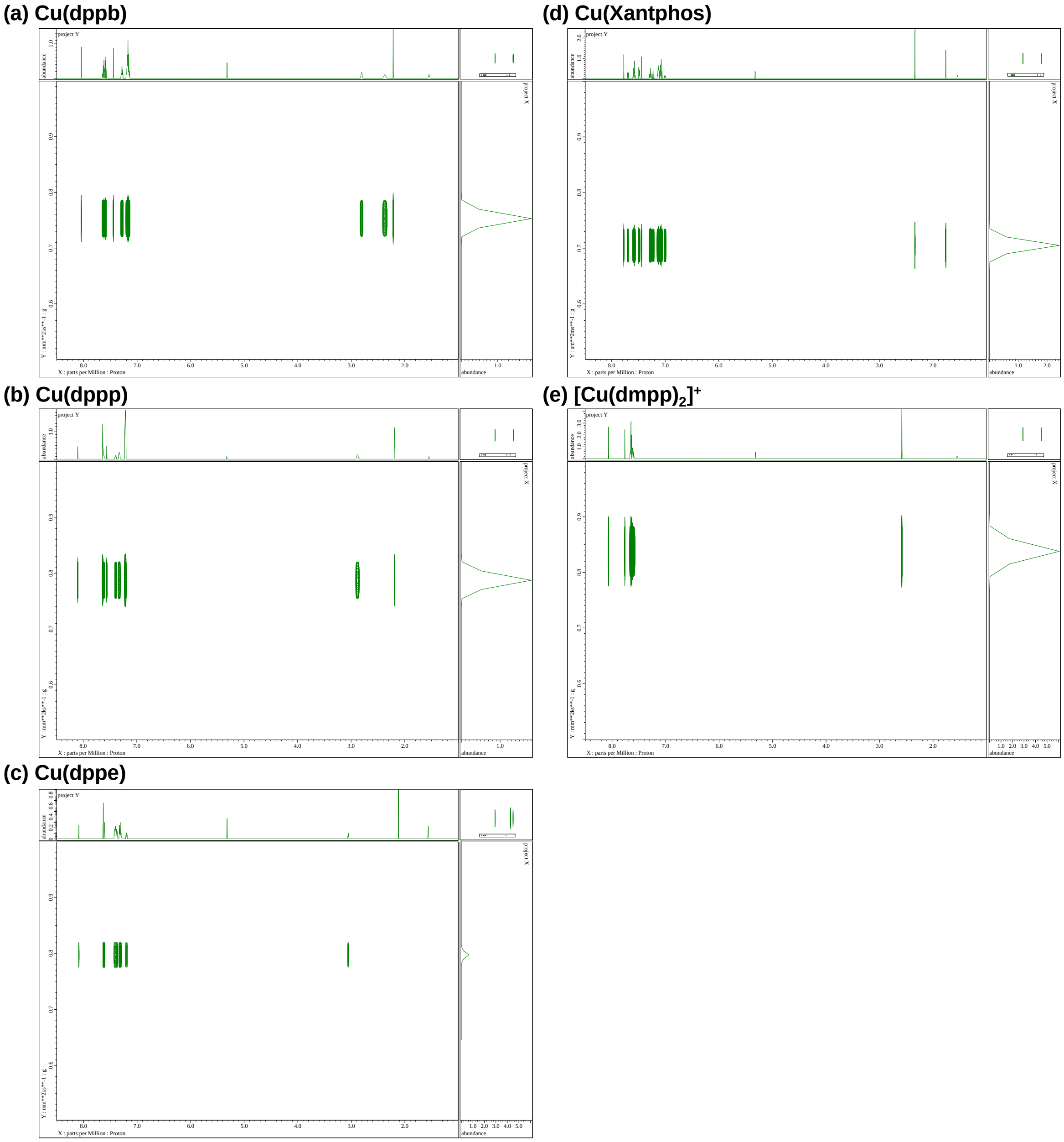

Figure S7. DOSY-NMR spectra of (a) **Cu(dppb)**, (b) **Cu(dppp)** (c) **Cu(dppe)**, (d) **Cu(Xantphos)**, (e) $[Cu(dmpp)_2](PF_6)$ measured in CD_2Cl_2.

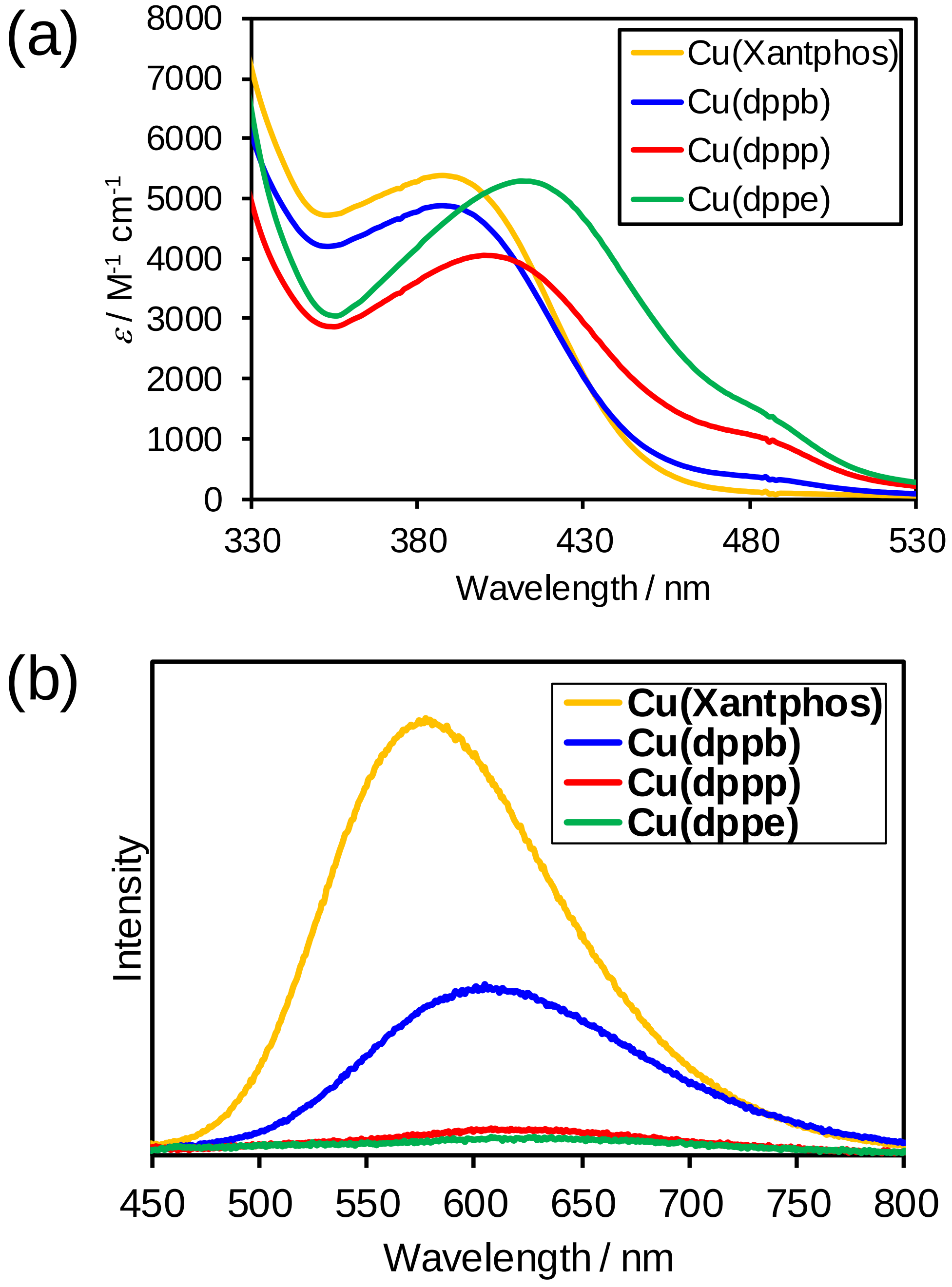

Figure S8. (a) UV-Vis absorption spectra and (b) emission spectra of the Cu(I) complexes (blue: **Cu(dppb)**, red: **Cu(dppp)**, green: **Cu(dppe)**, orange: **Cu(Xantphos)**). The solvent was a mixture of MeCN-TEOA (4:1 v/v). The excitation wavelength was 390 nm (**Cu(dppb)** and **Cu(Xantphos)**), 400 nm (**Cu(dppp)**) or 410 nm (**Cu(dppe)**) and the concentration of the Cu(I) complexes was 0.1 mM.

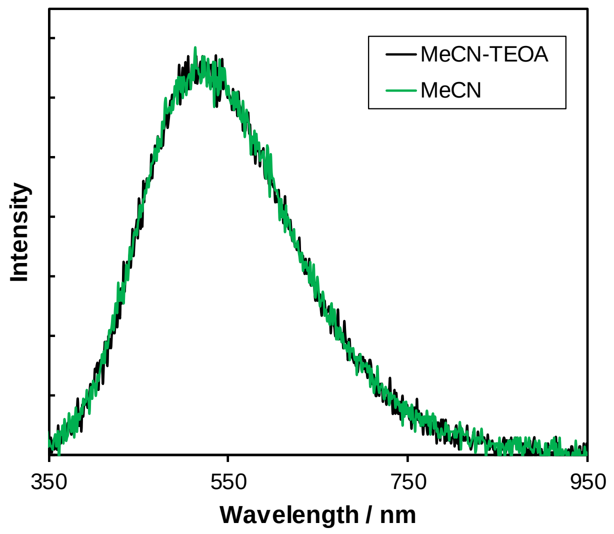

Figure S9. Emission spectra of 0.1 mM **Cu(dppb)** under irradiation at λ_{ex} = 390 nm. The solvent was MeCN (green) or MeCN-TEOA (4:1 v/v, black).

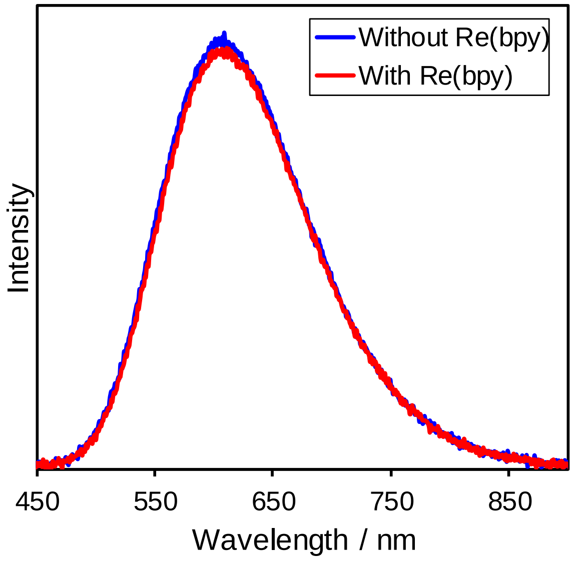

Figure S10. Emission spectra of 0.5 mM **Cu(dppb)** in the presence of 0.05 mM **Re(bpy)** (red) or in the absence of **Re(bpy)** (blue) under irradiation at λ_{ex} = 430 nm. The solvent was a mixture of MeCN-TEOA (4:1 v/v).

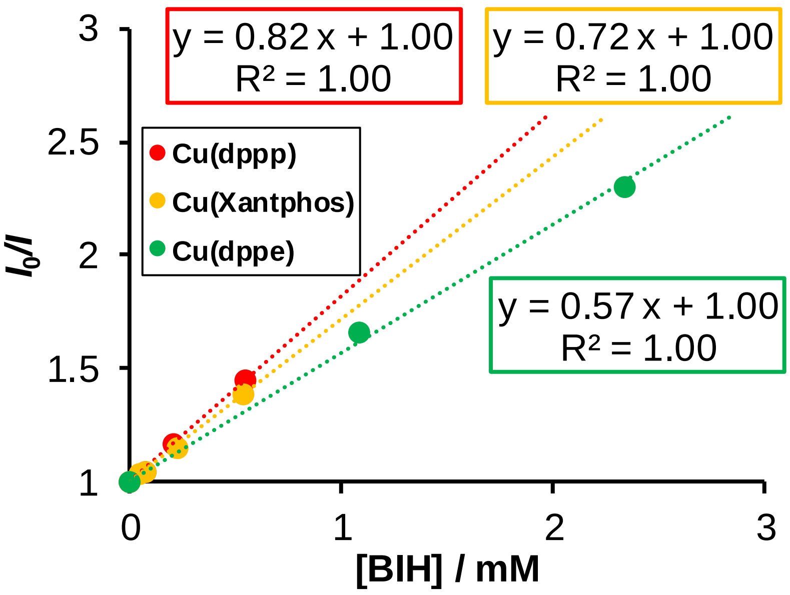

Figure S11. The Stern-Volmer plots using emission intensity (I) and the emission intensity without any BIH (I_0) (red: **Cu(dppp)**, green: **Cu(dppe)**, orange: **Cu(Xantphos)**). The solvent was a mixture of DMA-TEOA (4:1 v/v). Since the emission lifetimes of **Cu(dppp)**, **Cu(dppe)** and **Cu(Xantphos)** in the absence of BIH were 650 ns, 450 ns and 240 ns, respectively, the quenching rate constants were respectively determined to be 1.3×10^9 M^{-1} s^{-1}, 1.3×10^9 M^{-1} s^{-1} and 2.9×10^9 M^{-1} s^{-1}.

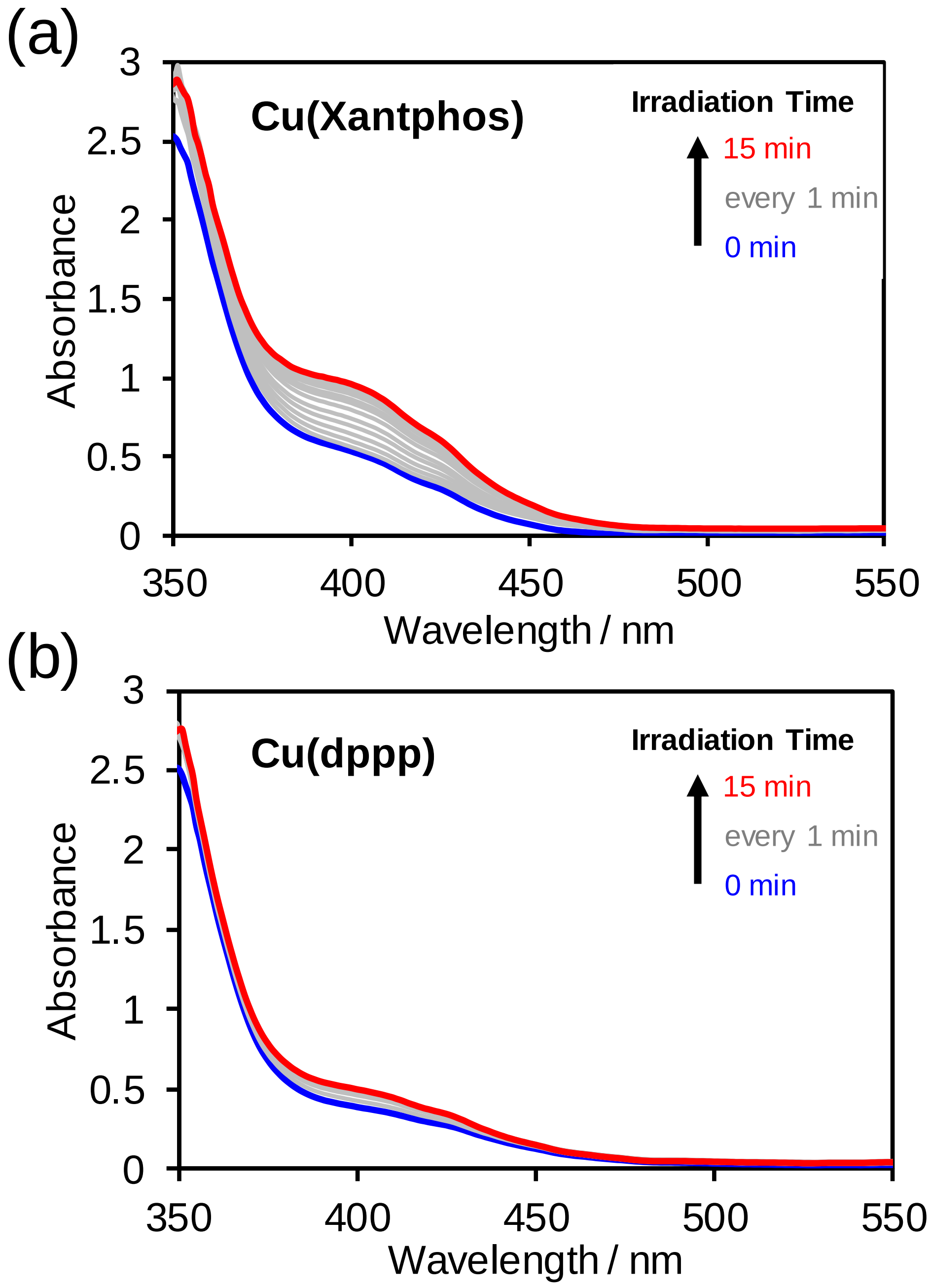

Figure S12. Absorption spectral changes of DMA–TEOA (4:1 v/v) solutions containing BIH (0.1 M) and 0.1 mM of (a) **Cu(Xantphos)** or (b) **Cu(dppp)**. The solution was irradiated with 430-nm monochromic light.

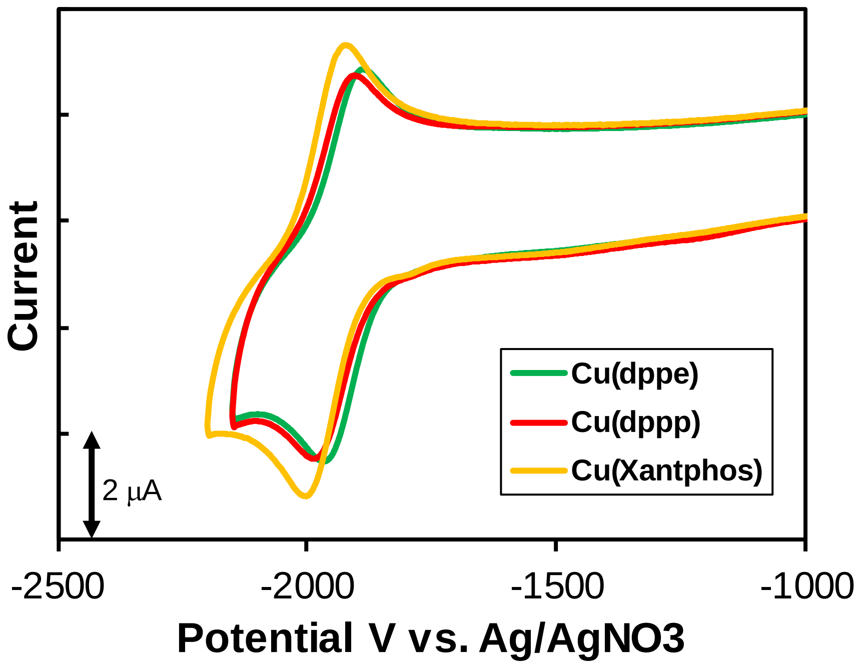

Figure S13. Cyclic voltammograms of **Cu(dppp)** (red), **Cu(dppe)** (green) or **Cu(Xantphos)** (orange) in a DMA-TEOA mixed solution (4:1 v/v) containing Et_4NPF_6 (0.1 M) as a supporting electrolyte with a $Ag/AgNO_3$ (0.01M) reference electrode. The concentration of each Cu complex was 0.5 mM.
